# MSRB7 reverses oxidation of GSTF2/3 to confer tolerance of *Arabidopsis thaliana* to oxidative stress

**DOI:** 10.1093/jxb/eru270

**Published:** 2014-06-24

**Authors:** Shu-Hong Lee, Chia-Wen Li, Kah Wee Koh, Hsin-Yu Chuang, Yet-Ran Chen, Choun-Sea Lin, Ming-Tsair Chan

**Affiliations:** ^1^Institute of Biotechnology, National Cheng Kung University, Tainan, 701, Taiwan; ^2^Academia Sinica Biotechnology Center in Southern Taiwan, Tainan, 741, Taiwan; ^3^Agricultural Biotechnology Research Center, Academia Sinica, Taipei, 115, Taiwan; ^4^Department of Biotechnology, TransWorld University, Douliu City, Yunlin County, 640, Taiwan

**Keywords:** *A. thaliana*, glutathione transferase, LC-MS/MS, methyl viologen, oxidative stress, methionine sulfoxide reductase B (MSRB).

## Abstract

Methionine sulfoxide reductases (MSRs) catalyse the reduction of oxidized methionine residues, thereby protecting proteins against oxidative stress. Accordingly, MSRs have been associated with stress responses, disease, and senescence in a taxonomically diverse array of organisms. However, the cytosolic substrates of MSRs in plants remain largely unknown. Here, we used a proteomic analysis strategy to identify MSRB7 substrates. We showed that two glutathione transferases (GSTs), GSTF2 and GSTF3, had fewer oxidized methionine (MetO) residues in *MSRB7*-overexpressing *Arabidopsis thaliana* plants than in wild-type plants. Conversely, GSTF2 and GSTF3 were highly oxidized and unstable in *MSRB7*-knockdown plants. MSRB7 was able to restore the MetO-GSTF2_M100/104_ and MetO-GSTF3_M100_ residues produced during oxidative stress. Furthermore, both GSTs were specifically induced by the oxidative stress inducer, methyl viologen. Our results indicate that specific GSTs are substrates of MSRs, which together provide a major line of defence against oxidative stress in *A. thaliana*.

## Summary

GSTF2/3 was identified to be a substrate of MSRB7 using our established CNBr digestion-based proteomic analysis, and the restoration of GSTF2/3 activity by MSRB7 was required for oxidative stress tolerance.

## Introduction

Reactive oxygen species (ROS), such as H_2_O_2_ and superoxide, are involved in signal transduction and defence mechanisms, although excess ROS can damage macromolecules, such as proteins, DNA, RNA, carbohydrates, and lipids (Moller and Sweetlove, 2010). This oxidative damage can lead to cell injury and even cell death. In the case of amino acids, both the free molecules and residues in polypeptides are targets of attack by ROS (Dat *et al.*, 2000; Friguet, 2006), and in proteins, this oxidation can alter protein conformation and function. The methionine (Met) residues of proteins are particularly susceptible to ROS-mediated oxidation, which results in the formation of two diastereoisomeric forms of methionine sulfoxide, Met-*S*-sulfoxide (Met-*S*-O) and Met-*R*-sulfoxide (Met-*R*-O). These oxidized forms of Met (MetO) can alter the conformation of a protein and render it non-functional. Therefore, the sensitivity of a protein to oxidative stress is related to the number of constituent Met residues.

Methionine sulfoxide reductases A (MSRA) and MSRB, which are found in many organisms, can reduce Met-*S*-O and Met-*R*-O, respectively (Vogt, 1995; Sharov and Schoneich, 2000), thus restoring the functional states of non-functional oxidized proteins. MSRs are, therefore, integral parts of an important protein repair system that protects organisms against oxidative stress (Oien and Moskovitz, 2008). The phylogenetic relationships and subcellular locations of MSRA and MSRB enzymes in *Arabidopsis thaliana* have been reported (Rouhier *et al.*, 2006; Tarrago *et al.*, 2009*a*), and genomic analyses have revealed the presence of nine *A. thaliana MSRB* genes. Proteins derived from two of the genes, *MSRB1* and *MSRB2*, are predicted to be chloroplastic, whereas MSRB3 is predicted to be localized to the secretory pathway and is translocated to the endoplasmic reticulum; the six remaining MSRBs are likely to be cytosolic (Rouhier *et al.*, 2006). The expressions of several *A. thaliana MSR* genes are modulated by abiotic stresses, including cold and high salinity, and by phytohormones such as abscisic acid (Oh *et al.*, 2005). For example, *MSRA4* is highly induced by high-light intensity or oxidative stress inducers such as methyl viologen (MV) and ozone, and its expression reduces the intracellular content of Met-*S*-O and confers protection against oxidative stress (Romero *et al.*, 2004). In addition, *A. thaliana msrb3* mutant accumulates more MetO and ROS than wild-type plants, independent of low temperature (Kwon *et al.*, 2007), whereas the *msrb1/msrb2* double mutant shows retarded growth and development under high-light and low-temperature conditions. The plastidial MSRBs are essential for maintaining plant growth through protection of the photosynthetic antennae (Laugier *et al.*, 2010), and transgenic tomato (*Solanum lycopersicum*) constitutively expressing the pepper (*Capsicum annuum*) *MSRB2* gene (*CaMSRB2*) was reported to have lower levels of ROS and enhanced resistance to pathogens (Oh *et al.*, 2010). Finally, the MSR repair system has been reported to establish and preserve longevity in seeds (Chatelain *et al.*, 2013).

Heat-shock protein 21 (HSP21) was the first specific substrate of plastidial MSRA identified in plants, and was shown to be crucial for plant resistance to oxidative stress. MSRA maintains the chaperone activity of HSP21 through the regeneration of the sulfoxidized N-terminal region, which contains a high proportion of Met residues (Gustavsson *et al.*, 2002). Recently, 24 proteins that interact with the *A. thaliana* plastidial MSRB1 were isolated by affinity chromatography (Tarrago *et al.*, 2012) and shown to be involved in photosynthesis, translation, and oxidative stress tolerance. Significantly, all of these interacting proteins have surface-exposed Met residues and higher-than-average Met contents, suggesting that they are more susceptible to oxidation by ROS and are dependent on plastidial MSRBs for repair (Tarrago *et al.*, 2012). However, no substrate of the cytosolic MSRB family in plants has been identified to date.

Our previous study indicated that *A. thaliana* overexpressing *MSRB7* (At4g21830) has a higher glutathione *S*-transferase (GST) activity and enhanced tolerance to oxidative stress (Li *et al.*, 2012). GSTs have been shown to be important for maintaining redox homeostasis, reducing oxidative damage (Cummins *et al.*, 1999), and protecting organisms against oxidative stress (Edwards and Dixon, 2005; Dixon *et al.*, 2011; Chen *et al.*, 2012). Accordingly, plant and animal GSTs are induced by various environmental stimuli, such as chilling, hypoxic stress, dehydration, wounding, pathogen attack, phytohormones and oxidative stress (Mauch and Dudler, 1993; Hayes *et al.*, 2005; Sappl *et al.*, 2009). In *A. thaliana* specifically, GSTs can be divided into seven classes: phi (F), tau (U), theta (T), zeta (Z), lambda (L), dehydroascorbate reductase and TCHQD (Dixon and Edwards, 2010), and can function as glutathione (GSH) transferases, GSH-dependent peroxidases, GSH-dependent isomerases, or GSH-dependent oxidoreductases (Edwards and Dixon, 2005). GSTs of stress-inducible plants may possess GSH-dependent peroxidase activities that act directly on H_2_O_2_, and at the same time are capable of utilizing GSH to reduce the organic hydroperoxides of fatty acids and nucleic acids (Dixon *et al.*, 2002). In addition, *A. thaliana* lacking GSTF2 shows increased sensitivity to MV and HgCl_2_ treatments (Gong *et al.*, 2005). These findings suggest an involvement of GSTFs in oxidative stress tolerance.

To investigate whether GSTs are the specific substrates of cytosolic MSRB7 under oxidative stress and to establish the mechanisms underlying the oxidative stress defence pathway, MSRB7 was subjected to functional analysis using proteomic, biochemical, and transgenic approaches.

## Materials and methods

### Plant materials and growth conditions

*A. thaliana* Heynh. ecotype Columbia plants were grown in controlled-environment chambers at 22 °C, 70% relative humidity, with a 16h photoperiod (approximately 120 μmol m^–2^ s^–1^). The floral dip transformation method (Clough and Bent, 1998) was used to generate transgenic *A. thaliana* lines. Ten-day-old seedlings were used for all experiments. For MV-tolerance experiments, plants were germinated and grown on Murashige and Skoog medium [4.3g Murashige and Skoog salt (Duchfa, Biochemie, Netherlands), Murashige and Skoog vitamins, 1% sucrose, 0.5g l^–1^ of MES, pH 5.7, 0.4% agar gel (Sigma-Aldrich, St Louis, MO, USA)] containing 10 μM MV. For protein stability experiments, plants were pre-treated with 10 μM MV for 8h, followed by 0.5mM cycloheximide (CHX) treatment for up to 36h.

### RNA isolation and gene expression analysis

Total RNA was isolated from plant tissues using Trizol reagent according to the manufacturer’s instructions (Invitrogen, Carlsbad, CA, USA). For reverse transcriptase (RT)-PCR, the cDNA was synthesized using a First-strand cDNA Synthesis kit (Promega, Madison, WI, USA). All gene-specific primers are listed in Supplementary Table S2 available at *JXB* online. Real-time PCR amplification was performed using SYBR Green Master Mix (Applied Biosystems, Foster City, CA, USA) and monitored using an ABI 7500HT sequence detection system (Applied Biosystems). Data were analysed using ABI SDS 1.4 software (Applied Biosystems). Relative transcript levels were normalized to expression of the endogenous control genes *Actin2* (At3g18780), *EF1α* (At5g60390), and *18S* rRNA (AF206999) using the comparative cycle threshold (*C*_t_) method.

### Plasmid constructions

The full-length *MSRB7*-encoding gene was isolated by RT-PCR (Supplementary Table S2 available at *JXB* online) from 2-week-old *A. thaliana* seedlings and subcloned into the binary vector pCAMBIA1390:35S (*B7*Ox) (Hsiao *et al.*, 2007). The 5′ region including the 5′-untranslated region (5′-UTR) and partial coding sequence of *MSRB7* was cloned into the pH7GWIWG vector (Invitrogen) to generate RNA interference knockdown plants (*B7*i) (Li *et al.*, 2011). For β-glucuronidase (GUS) histochemical staining, the *MSRB7* promoter region (2000bp upstream of the start codon) was cloned into the pHGWFS7 vector (Invitrogen) to regulate the expression of the *GUS*-encoding gene. These plasmids were transformed into *Agrobacterium tumefaciens* strain GV3101 (pMP90) by electroporation for use in *A. thaliana* transformation.

### GUS histochemical staining and activity

*MSRB7* promoter-driven *GUS* (*B7pro-GUS*), pCAMBIA1301 transgenic plants (*CaMV35Spro-GUS*; 1301), and wild-type plants were examined histochemically using GUS staining (0.1M sodium phosphate buffer, pH 7.0, 10mM EDTA, 0.5mM potassium ferrocyanide, 0.5mM potassium ferricyanide, 0.1% Triton X-100, 1mM 5-bromo-4-chloro-3-indolyl-β-d-glucuronic acid). GUS activity was determined as described previously (Lu *et al.*, 1998).

### Comparative proteomic analysis using cyanogen bromide (CNBr) digestion

Cytosolic proteins were extracted from 10-d-old MV-treated seedlings using ice-cold buffer consisting of 50mM HEPES (pH 7.5), 300mM sucrose, 150mM NaCl, 10mM potassium acetate, 5mM EDTA, 1mM phenylmethylsulfonyl fluoride (PMSF), and 1× Protease Inhibitor Cocktail (Sigma-Aldrich). Proteins were digested overnight with 100mM CNBr in 50% trifluoroacetic acid in the dark at room temperature (Ogorzalek Loo *et al.*, 1996), followed by trypsin digestion. Peptide mass fingerprinting was performed as described previously (Chen *et al.*, 2010). Liquid chromatography tandem mass spectrometry (LC-MS/MS) was performed with a nanoflow LC system (nanoACQUITY UPLC; Waters, Millford, MA, USA) coupled to a hybrid Q-TOF mass spectrometer (Synapt HDMS G2; Waters, Manchester, UK). For the nanoflow LC system, mobile phase A contained water with 0.1% formic acid and phase B contained acetonitrile with 0.1% formic acid. The peptide samples were injected onto a trap column (Symmetry C18, 5 μm, 180 μm×20mm; Waters, Milford, MA, USA) and separated online with a reverse-phase column (BEH C18, 1.7 μm, 75 μm×250mm; Waters, Milford, MA, USA) at a flow rate of 300 nl min^–1^ using a 90min 15–90% acetonitrile/water gradient. The temperature of the separating column was maintained at 35 °C. For MS analysis, the LC column was online-coupled to the nanospray source of a hybrid Q-TOF mass spectrometer and 500fmol μl^–1^ of [Glu]fibrinopeptide B was continuously infused to the lockspray emitter at a flow rate of 250 nl min^–1^. The MS was switched to the lockspray source every 30 s and the [Glu]fibrinopeptide signal was used as a reference mass for calibration. The LC-MS data were collected in MS^E^ mode: the low collision energy spectra were acquired at 4eV trapping energy and the high collision energy spectra were acquired by ramping the trapping energy from 10 to 30eV. The low and high collision energy scan range was from 50 to 1990 Th with a scan time of 1 s and a 0.02 s interscan time. The MS^E^ data were processed using the ProteinLynx GlobalServer (PLGS, version 2.3; Waters, Manchester, UK). To process the chromatogram of each precursor and fragment ion, the minimal peak width was subjected to three scans and the expected peak was subjected to seven scans. The spectral noise was processed using the adaptive background subtraction provided by PLGS, and the maximum charge state for deisotoping was 6. For database searching, the IPI ARATH v.3.85 FASTA database (ftp://ftp.ebi.ac.uk/pub/databases/IPI) was used. CNBr and trypsin were used specifically as the digestion reagents, one missed cleavage was allowed, carbamidomethyl (C) was specified as the fixed modification, and oxidation (M) was considered as a variable modification. Peptides were considered identified if the identification confidence value was >95% in PLGS. Each peptide identified was further quantified by the Expression^E^ tool in PLGS. All the quantified peptides were used to calculate the relative abundance of the proteins, and the protein abundance ratios were finally normalized by the ‘auto normalization’ function of PLGS.

### Met residues in GSTs and their differential oxidation as analysed by MS

Cytosolic proteins were extracted by ice-cold cytosolic protein extraction buffer [PBS containing 5mM EDTA, 1mM PMSF, 1mM dithiothreitol (DTT), 1× Protease Inhibitor Cocktail (Sigma-Aldrich), 10% glycerol, and 0.01% Tween 20]. Protein samples were separated by SDS-PAGE. Proteins in the size range 25–30kDa were digested by trypsin and then analysed by LC-MS/MS. The MS^E^ data were processed using PLGS. The amino acid sequences of GSTF2, GSTF3, and GSTF8 were referred for database searching. Coverage represents the number of times the peptide containing Met residues, both oxidized and reduced forms, was detected. The percentage of oxidization was calculated using the following formula: % oxidization=[number of MetO on GSTF2_M100_ / number of both oxidized and reduced forms of Met on GSTF2_M100_]×100.

### Immunoblot analysis

Cytosolic proteins were extracted using cytosolic protein extraction buffer. Protein samples were separated by SDS-PAGE and electrotransferred to a polyvinylidene difluoride membrane. GSTF2, GSTF3, and MSRB7 were recognized with anti-GSTF2/3 antibody (Agrisera, Vännäs, Sweden) and in-house rabbit anti-MSRB7 antibody, respectively. Antibody-bound proteins were detected using a chemiluminescence system (Millipore Corporation, Billerica, MA, USA) following incubation with protein A-conjugated horseradish peroxidase (Invitrogen).

### Production of recombinant proteins

The Met residues on GSTF2 and GSTF3 were mutated by PCR using the primers for site-directed mutagenesis listed in Supplementary Table S2 available at *JXB* online. The *MSRB7* N terminus fused with a flag tag (*flag–MSRB7*), *GSTF2*, *GSTF3*, *GSTF8*, *GSTF2*_M100L_ (Met replaced by Leu), *GSTF2*_M104L_, *GSTF2*_M100/104L_, and *GSTF3*_M100L_ were cloned into the pET-53-DEST^TM^ vector (Merck KGaA, Darmstadt, Germany). These vectors were transformed into the *Escherichia coli* Rosetta (DE3) strain. The recombinant His–flag–MSRB7, His–GSTF2, His–GSTF3, His–GSTF8, His–GSTF2_M100L_, His–GSTF2_M104L_, His–GSTF2_M100/104L_, and His–GSTF3_M100L_ proteins were purified using Ni^2+^-affinity columns. Recombinant MSRB7 proteins were detected using anti-flag or anti-His antibodies (Sigma-Aldrich).

### Bimolecular fluorescence complementation (BiFC) and protoplast transient assay

The full-length coding regions of *MSRB7*, *GSTF2*, *GSTF3*, and *GSTF8* were cloned into BiFC vectors (Supplementary Fig. S1 available at *JXB* online) (Walter *et al.*, 2004). Protoplasts were isolated using the tape–*A. thaliana* sandwich method and co-transformed with plasmid expressing nuclear-localizing marker [(bZIP63–CFP (cyan fluorescent protein)] (Walter *et al.*, 2004) and BiFC plasmids using the polyethylene glycol method (Wu *et al.*, 2009). After incubation at room temperature for 16h under light, the protoplasts were observed with a Zeiss LSM510 META laser scanning confocal microscope (Carl Zeiss, Jena, Germany).

### Yeast two-hybrid assay

The ProQuest two-hybrid system (Invitrogen) was used in a yeast two-hybrid assay. *MSRB7* was cloned into pDEST22 as bait, and *GSTF2*, *GSTF3*, and *GSTF8* were cloned into pDEST32 as prey. The construct pairs were co-transformed into yeast strain MaV203 according to the manufacturer’s instructions (Invitrogen). Positive yeast transformants were selected on SD minimal (–Leu–Trp) and (–Leu–Trp–His) medium and experiments were performed with three biological repeats. Appropriate controls were included by co-transforming pEXP32/Krev1 with pEXP22/ RalGDs-WT (wild type with strong interaction), pEXP22/RalGDs-m1 (mutant with weak interaction), and pEXP22/RalGDs-m2 (mutant with no interaction).

### GST activity

For *in vitro* GST activity assays, recombinant GST protein was oxidized by treatment with 0.5mM hypochlorous acid (HOCl) for 40min at room temperature and a final concentration of 5mM Met was added to terminate the oxidative reaction. Sixty micrograms of oxidant-treated GST was co-incubated with 20 μg of MSRB7 in PBS containing 10mM MgCl_2_, 30mM KCl, and 10mM DTT for 1h at room temperature. The activity of recombinant GST was measured using a 1-chloro-2,4-dinitrobenzene (CDNB) assay as described previously (Habig *et al.*, 1974). For *in vivo* GST activity assay, 10-d-old *B7*Ox, *B7*i, and 1301 seedlings were treated with 10 μM MV for 8h and then treated with CHX for 0, 12, 24, and 36h. The CHX-treated *A. thaliana* proteins were extracted using cytosolic protein extraction buffer and the GST activity was then measured using a CDNB assay.

### Immunoprecipitation

His–flag–MSRB7, oxidized His–GSTF2, oxidized His–GSTF3, and oxidized His–GSTF8 recombinant proteins were incubated with rabbit anti-flag antibody in IP buffer (15mM HEPES, pH 7.4, 1mM EDTA, 1mM DTT, 10mM MgCl_2_, 30mM KCl, 1mM PMSF, and 1× Protease Inhibitor Cocktail) for 1h at room temperature. Protein G–Sepharose beads (Invitrogen) were added and the samples were incubated for 1h at room temperature for co-precipitation. The immunoprecipitate was immunoblotted and detected using mouse anti-His antibody.

### H_2_O_2_ content

Ten-day-old *A. thaliana* plants treated with 10 μM MV for 24h were extracted by PBS. H_2_O_2_ content was measured using anAmplex Red Hydrogen Peroxide/Peroxidase assay kit (Invitrogen), according to the manufacturer’s instructions.

### Statistical analyses

Data are presented as mean values±standard deviation (SD). Duncan’s test was performed to calculate the differences between distributions of data using SPSS v.12.0 software. *P* values of less than 0.05 were considered statistically significant.

## Results

### Identification of putative MSRB7 substrates by comparative proteomic analysis using CNBr digestion

**Fig. 1.**
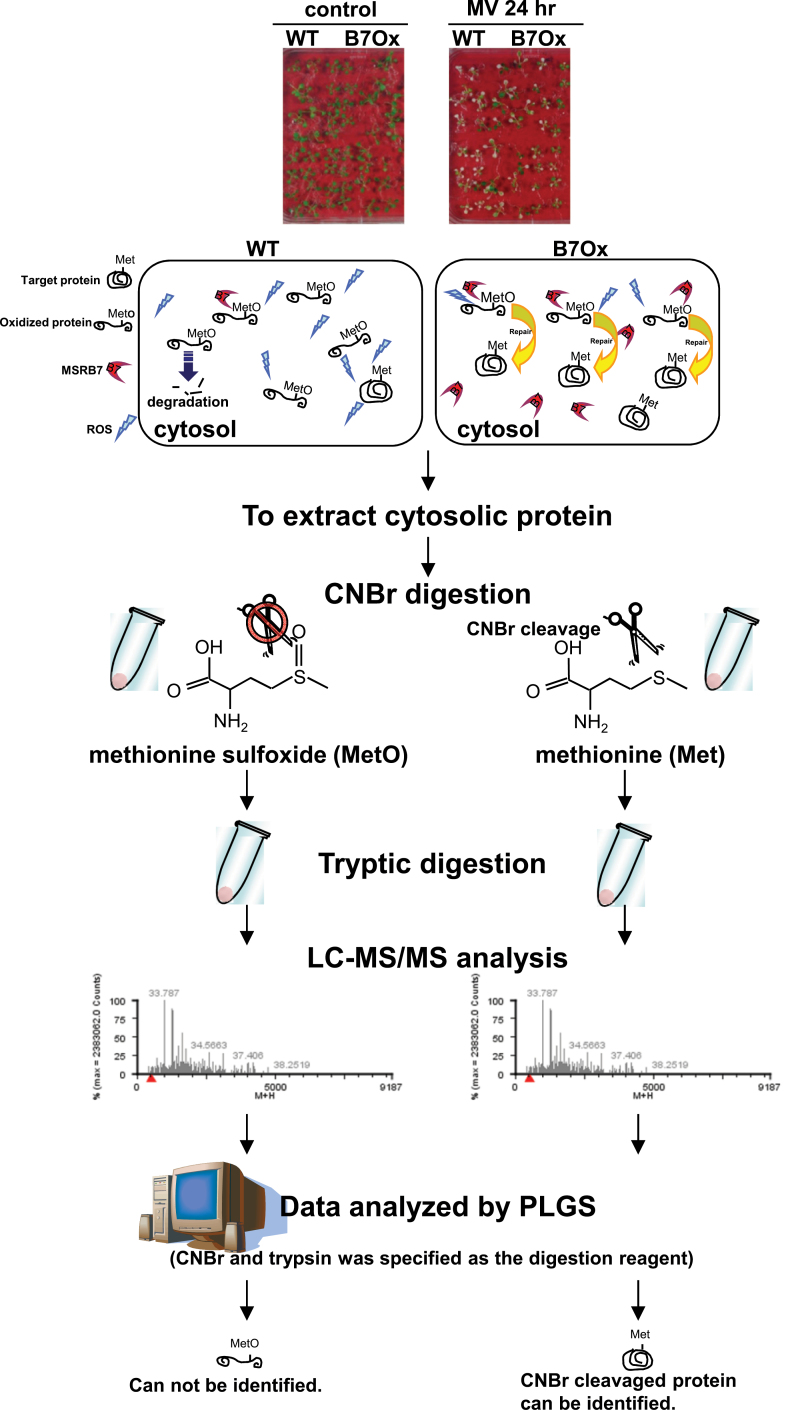
Flowchart of the steps used for comparative proteomic analysis using the CNBr digestion approach.

To identify the substrates of MSRB7, cytosolic protein-enriched extracts were isolated from *B7*Ox and wild-type plants, digested with CNBr and trypsin, and analysed using LC-MS/MS. CNBr specifically hydrolyses the C terminus of Met but not MetO residues, and therefore proteins harbouring MetO residues are not hydrolysed by CNBr. A flowchart of the steps used for the comparative proteomic analysis using the CNBr digestion approach is presented in Fig. 1. The CNBr/trypsin-digested samples were first subjected to LC-MS/MS analysis, and the MS^E^ data obtained were processed using the PLGS, with CNBr and trypsin specified as the digestion reagents for the database analysis search (Fig. 1). This strategy was designed to allow the identification of proteins/peptides that contained Met, as opposed to MetO, as a consequence of MSRB7 activity, and were therefore amenable to CNBr digestion, identification, and quantification (Fig. 1). This analysis led to the identification of a total of 188 such proteins (Supplementary Table S1 available at *JXB* online). To identify the possible interacting partners of MSRB7, putative targets that were present only in the proteomic data from the *B7*Ox sample, or were >1.5 fold higher in *B7*Ox than in the wild type, were selected for further analysis. Analysis of the Gene Ontology (GO) annotations in The Arabidopsis Information Resource (TAIR; (http://www.arabidopsis.org/tools/bulk/go/index.jsp) corresponding to these proteins indicated that 41 of the putative targets were related to stress responses, some of which, such as GST, peroxidase, and catalase, are known to be involved in ROS scavenging (Table 1). GSTF2 (At4g02520), GSTF3 (At2g02930), and GSTF8 (At2g47730) were more abundant in *B7*Ox than the wild type, suggesting that they may be interacting partners of MSRB7, and since GST activity was higher in the *MSRB7*-overexpressing plants than in the wild-type plants (Li *et al.*, 2012), we hypothesized that GSTF2, GSTF3, and GSTF8 are direct substrates of MSRB7.

**Table 1.** Potential substrates of MSRB7 Ten-day-old *B7*Ox and wild-type *A. thaliana* were treated with or without 10 μM MV for 24h. ND, not determined.

Accession no.	Locus	Description	Score	*B7*Ox/WT MV 24h	*B7*Ox/WT MV 0 h	Unique
IPI00537995	At1g35720	Annexin D1	218.40	*B7*Ox 24h	*B7*Ox 0h	*B7*Ox only
IPI00535149	AT4g02520	Glutathione S transferase F2	188.83	*B7*Ox 24h	*B7*Ox 0h	*B7*Ox only
IPI00525727	At4g37930	Mitochondrial, serine hydroxymethyltransferase mitochondrial	78.56	*B7*Ox 24h	*B7*Ox 0h	*B7*Ox only
IPI00523477	At5g38420	Chloroplastic, ribulose bisphosphate carboxylase small chain 2B	2906.54	*B7*Ox 24h	3.03	
IPI00532772	At1g66200	Glutamine synthetase cytosolic isozyme 1	254.75	*B7*Ox 24h	1.47	
IPI00532945	At2g02930	Glutathione *S*-transferase F3	170.04	*B7*Ox 24h	ND	*B7*Ox 24h
IPI00520226	At4g14960	Tubulin α6 chain	261.54	*B7*Ox 24h	ND	*B7*Ox 24h
IPI00530621	At1g19570	Dehydroascorbate reductase 1 (DHAR1)	155.99	*B7*Ox 24h	1.00	
IPI00544626	At3g01500	Chloroplastic, isoform 1 of carbonic anhydrase	756.83	*B7*Ox 24h	1.00	
IPI00534087	At5g56010	Heat-shock protein 81 3	203.99	2.38	*B7*Ox 0h	
IPI00533497	At3g09260	β-Glucosidase	102.90	1.43	*B7*Ox 0h	
IPI00544876	At3g55800	Chloroplastic, sedoheptulose 1,7 bisphosphatase	111.61	1.41	*B7*Ox 0h	
IPI00539020	At1g67090	Chloroplastic, ribulose bisphosphate carboxylase small chain 1A	3213.8	1.63	1.59	
IPI00521186	At5g38430	Chloroplastic, ribulose bisphosphate carboxylase small chain 1B	2947.88	1.05	2.30	
IPI00656928	At4g35090	Catalase 2	108.7	0.76	1.54	
IPI00532582	At4g21280	Isoform 2 of oxygen evolving enhancer protein 3	296.93	0.75	*B7*Ox 0h	
IPI00891841	At5g38410	Similar to ribulose bisphosphate carboxylase small chain 2B	2291.48	ND	*B7*Ox 0h	*B7*Ox 0h
IPI00532125	At1g54040	Epithiospecifier protein	596.6	ND	*B7*Ox 0h	*B7*Ox 0h
IPI00846574	At5g14740	β-Carbonic anhydrase 2	330.81	ND	*B7*Ox 0h	*B7*Ox 0h
IPI00518163	At2g39730	Ribulose bisphosphate carboxylase oxygenase activase	291.06	ND	*B7*Ox 0h	*B7*Ox 0h
IPI00542532	At1g24020	MLP-like protein 423	245.59	ND	*B7*Ox 0h	*B7*Ox 0h
IPI00516423	At4g25050	Acyl carrier protein 4	193.68	ND	*B7*Ox 0h	*B7*Ox 0h
IPI00518090	At1g13440	Glyceraldehyde-3-phosphate dehydrogenase C2 (GAPC2)	190.2	ND	*B7*Ox 0h	*B7*Ox 0h
IPI00656779	At2g21330	Fructose bisphosphate aldolase	160.8	ND	*B7*Ox 0h	*B7*Ox 0h
IPI00518620	At3g32980	Peroxidase 32	147.69	ND	*B7*Ox 0h	*B7*Ox 0h
IPI00539116	At5g26000	Myrosinase	135.6	ND	*B7*Ox 0h	*B7*Ox 0h
IPI00524641	At2g21170	Chloroplastic, triosephosphate isomerase	127.79	ND	*B7*Ox 0h	*B7*Ox 0h
IPI00523903	At5g02490	Heat-shock cognate 70kDa protein 2	121.8	ND	*B7*Ox 0h	*B7*Ox 0h
IPI00536062	At2g47730	Glutathione *S*-transferase F8	117.32	ND	*B7*Ox 0h	*B7*Ox 0h
IPI00526611	At1g56410	Early response to dehydrogenase 2 (HSP70)	116.83	ND	*B7*Ox 0h	*B7*Ox 0h
IPI00547926	At3g18780	Actin 2	115.01	ND	*B7*Ox 0h	*B7*Ox 0h
IPI00538349	At1g63940	Monodehydroascorbate reductase 6	103.82	ND	*B7*Ox 0h	*B7*Ox 0h
IPI00539389	At1g16030	Heat-shock protein 70B (HSP70B)	96.9	ND	*B7*Ox 0h	*B7*Ox 0h
IPI00539339	At3g04790	Ribose 5-phosphate isomerase related	91.58	ND	*B7*Ox 0h	*B7*Ox 0h
IPI00545934	At5g12250	Tubulin β6 chain	84.79	ND	*B7*Ox 0h	*B7*Ox 0h
IPI00530539	At5g64290	Dicarboxylate transport 2	84.74	ND	*B7*Ox 0h	*B7*Ox 0h
IPI00525001	At5g62690	Tubulin β2β3 chain	82.8	ND	*B7*Ox 0h	*B7*Ox 0h
IPI00523675	At4g23210	Isoform 2 of cysteine rich receptor-like protein kinase 13	79.72	ND	*B7*Ox 0h	*B7*Ox 0h
IPI00518916	At5g24300	Chloroplastic amyloplastic, soluble starch synthase	78.84	ND	*B7*Ox 0h	*B7*Ox 0h
IPI00517585	At5g52250	Transducin family protein	76.69	ND	*B7*Ox 0h	*B7*Ox 0h
IPI00530974	At1g52770	Phototropic responsive NPH3 family protein	76.1	ND	*B7*Ox 0h	*B7*Ox 0h

### MSRB7, GSTF2, and GSTF3 are induced by MV

**Fig. 2.**
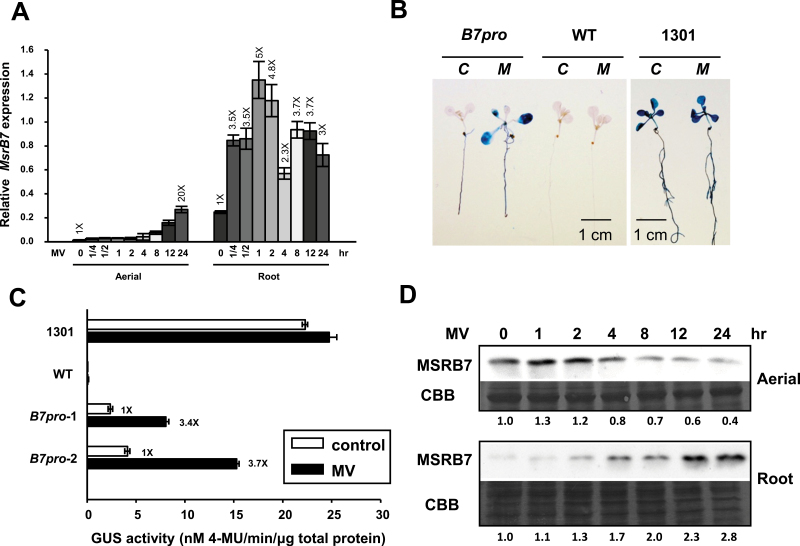
Induction of *MSRB7*, *GSTF2*, and *GSTF3* by oxidative stress. (A) Expression patterns of *MSRB7.* Real-time PCR analysis of transcripts of 10-d-old *A. thaliana* plants treated with 10 μM MV for 15min to 24h. The data represent the means±SD (*n*=10) of three independent experiments. (B, C) Histochemical GUS staining and GUS activity. *A. thaliana* seedlings harbouring the *MSRB7* promoter (*B7pro*)-driven *GUS* were untreated (*C*) or treated (*M*) with 10 μM MV for 8h and GUS expression (B) and activity (C) were determined. Wild-type (WT) and pCAMBIA1301 transgenic plants (*CaMV35Spro*-GUS; 1301) served as negative and positive controls, respectively. (D) Immunoblotting of MSRB7. Ten-d-old wild-type seedlings were treated with 10 μM MV for 0–24h and expression of the MSRB7 protein was detected with a specific anti-MSRB7 antibody. Protein stained with Coomassie Brilliant Blue (CBB) was used as a loading control.

Real-time PCR analysis was performed to investigate whether the expression of *MSRB7*, *GSTF2*, and/or *GSTF3* was affected by MV-induced oxidative stress. *MSRB7* transcripts were highly expressed in roots and were strongly induced by 10 μM MV, with transcript levels increasing gradually during the first 2h of MV treatment and remaining high for 12–24h post-treatment (Fig. 2A). Conversely, *MSRB7* transcripts were expressed at low levels in the aerial parts of plants, although prolonged MV treatment resulted in a gradual increase in transcript levels (Fig. 2A). We next examined the locations of *MSRB7* expression using transgenic *A. thaliana* lines transformed with a *GUS* gene driven by the *MSRB7* promoter (*B7pro-GUS*). Histochemical staining of 10-d-old transgenic seedlings indicated that *MSRB7* was expressed in roots but not shoots under normal conditions (Fig. 2B), confirming the PCR results. However, upon treatment with MV for 8h, GUS expression was highly induced throughout the whole seedling (Fig. 2B, C), suggesting that MV induces *MSRB7* expression. In addition, GUS staining of 6-week-old *B7pro-GUS* seedlings treated with MV revealed expression in the cauline and rosette leaves but not in the flowers or siliques (Supplementary Fig. S2 available at *JXB* online). Wild-type and transgenic plants transformed with the pCAMBIA1301 (*CaMV35Spro-GUS*; 1301) were used as negative and positive controls, respectively (Fig. 2B, C). Immunoblot analysis showed an increase in MSRB7 protein expression in the roots upon MV treatment (Fig. 2D), supporting the above observations (Fig. 2A, B). In the case of the aerial part, the MSRB7 protein abundance decreased upon MV treatment (Fig. 2D).

**Fig. 3.**
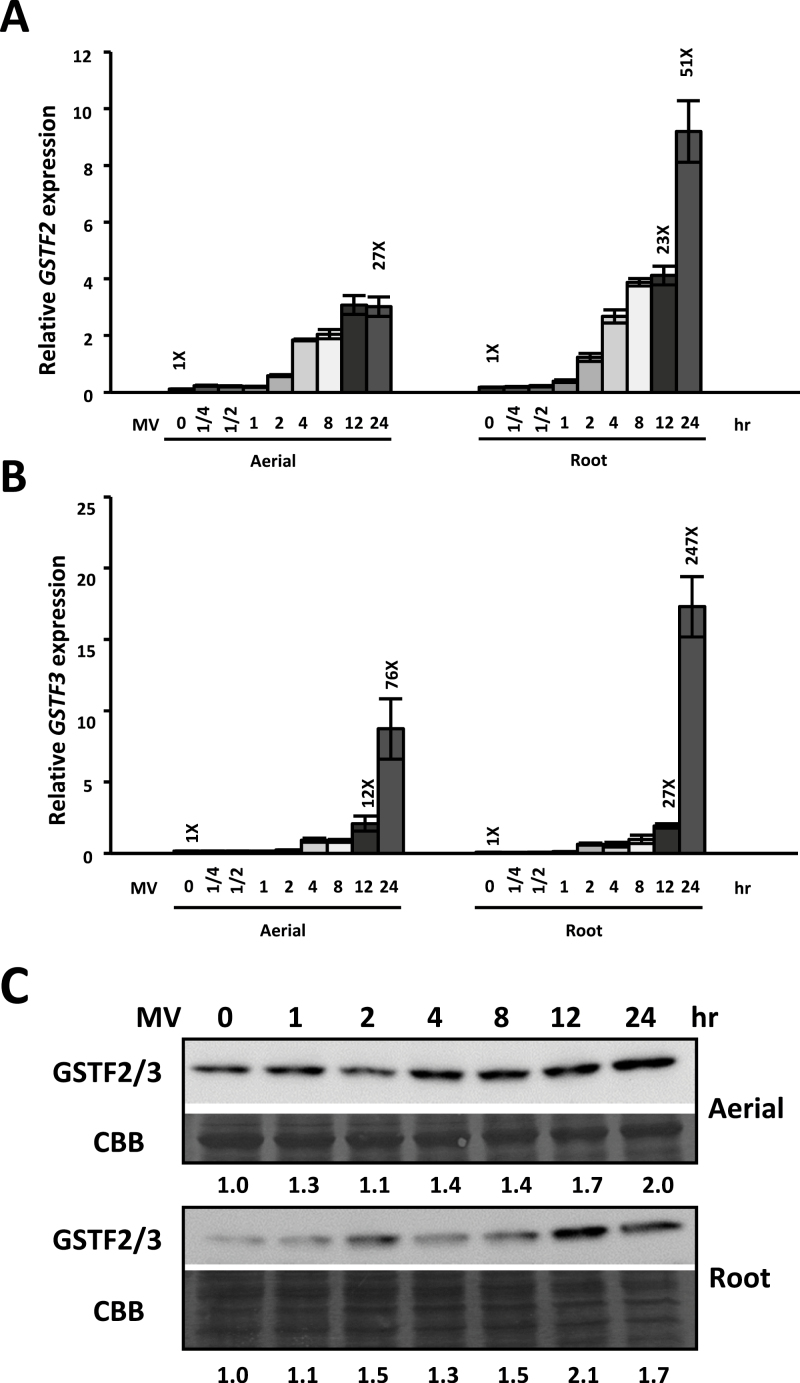
Induction of *GSTF2* and *GSTF3* by oxidative stress. (A, B) Expression patterns of *GSTF2* and *GSTF3*. Real-time PCR analysis of transcripts in 10-d-old *A. thaliana* plants treated with 10 μM MV for 15min to 24h. The data represent the means±SD (*n*=10) of three independent experiments. (C) Immunoblotting of GSTF2/3. Ten-d-old wild-type seedlings were treated with 10 μM MV for 0–24h. GSTF2/3 expression was detected using an anti-GSTF2/3 antibody. Protein stained with Coomassie Brilliant Blue (CBB) was used as a loading control.

Based on sequence similarities/identities, *GSTF2*, *GSTF3*, and *GSTF8* have been classified into the F class of the GST family (Dixon and Edwards, 2010). The polypeptides of GSTF2 and GSTF3, which are 92.5% identical with respect to their amino acid sequences, were both detected using the same GSTF2/3 antibody (Agrisera) (Supplementary Fig. S3 available at *JXB* online). MV treatment resulted in high expression levels and accumulation of *GSTF2* and *GSTF3* at both the transcript and protein levels, (Fig. 3); however, the expression pattern of *GSTF8* showed no apparent changes following MV treatment (Supplementary Fig. S3B available at *JXB* online), indicating that the expression of *GSTF2* and *GSTF3*, but not *GSTF8*, was MV inducible. The abundance of GSTF2/3 protein remained high in the aerial part of the plant, and gradually increased in the root part upon MV treatment, suggesting that these proteins, together with MSRB7, have a role in protecting plants against oxidative stress.

### Met residues in GSTF2 and GSTF3 are repaired by MSRB7 *in vivo*

To investigate the role of MSRB7 in the reducing of MetO residues in plant GSTF2 and GSTF3, MetO levels in the GSTs were quantified by MS. Ten-day-old *B7*Ox, *B7*i, and 1301 (vector-only control) plants were treated with MV for 24h, and protein samples from these and untreated plants were collected and subjected to tryptic digestion followed by LC-MS/MS analysis. The percentage of MetO was calculated from the identified peptides containing specific Met residues, and the coverage was defined as the number of times the peptide containing these Met residues was found (MetO and Met). In addition to the first Met residue, GSTF2, GSTF3, and GSTF8 contain two (GSTF2_M100/104_), one (GSTF3_M100_), and six (GSTF8_M49/59/67/84/173/176_) Met residues, respectively (Supplementary Fig. S4 available at *JXB* online). We observed that MV treatment resulted in an increase in the average percentage of MetO residues in all three proteins. In particular, GSTF2_M104_ and GSTF3_M100_ were susceptible to oxidization in *B7*i (34.5 and 60%, respectively) and 1301 (17.2 and 26.2%, respectively) plants under oxidative stress, and even in the absence of MV-induced oxidative stress, GSTF3_M100_ was highly oxidized (66.7%) in *B7*i plants. The percentages of MetO in GSTF2 and GSTF3 were significantly lower in *B7*Ox plants than in *B7*i and 1301 plants, while the MetO residues in GSTF8 were less frequently converted back to the reduced Met state in *B7*Ox plants (Table 2). These observations indicated that GSTF2 and GSTF3 may be direct substrates of MSRB7 in plants under oxidative stress.

**Table 2.** Met residues in GSTs and their differential oxidation as revealed by MS Ten-day-old *B7*Ox, *B7*i and 1301 plants were treated with or without 10 μM MV for 24h. Experiments were repeated three times. Results are shown as % oxidation. The values in brackets is the coverage, representing the number of times the peptide containing Met residues was found in both the oxidized and the reduced form, is shown in parentheses.

Met position	1301		*B7*Ox		*B7*i	
	Control	MV treated	Control	MV treated	Control	MV treated
**GSTF2**						
100	0 (21)	17.2 (29)	0 (24)	0 (44)	0 (17)	0 (81)
104	0 (21)	17.2 (29)	0 (24)	4.8 (44)	0 (17)	34.5(81)
Average	0 (21)	17.2 (29)	0 (24)	2.4 (44)	0 (17)	17.2 (81)
**GSTF3**						
100	0 (12)	26.2 (42)	0 (24)	16.1 (31)	66.7 (24)	60.0 (20)
Average	0 (12)	26.2 (42)	0 (24)	16.1 (31)	66.7 (24)	60.0 (20)
**GSTF8**						
49	0 (11)	0 (18)	0 (13)	0 (15)	0 (20)	16.7 (30)
59	0 (23)	24.1 (29)	0 (12)	22.6 (31)	0 (18)	33.3 (27)
67	0 (21)	0 (29)	0 (8)	22.6 (31)	16.7 (30)	28.6 (21)
84	0 (28)	36.1 (108)	0 (14)	33.9 (124)	7.0 (43)	46.2 (184)
173	25.9 (27)	44.7 (94)	0 (7)	40.4 (73)	14.6 (41)	36.3 (113)
176	0 (38)	44.7 (94)	0 (12)	17.6 (85)	0 (49)	27.0 (152)
Average	4.3 (24.7)	24.9 (62)	0 (11)	22.9 (59.8)	6.4 (33.5)	31.3 (87.8)

### GSTF2, GSTF3, and GSTF8 interact with MSRB7

**Fig. 4.**
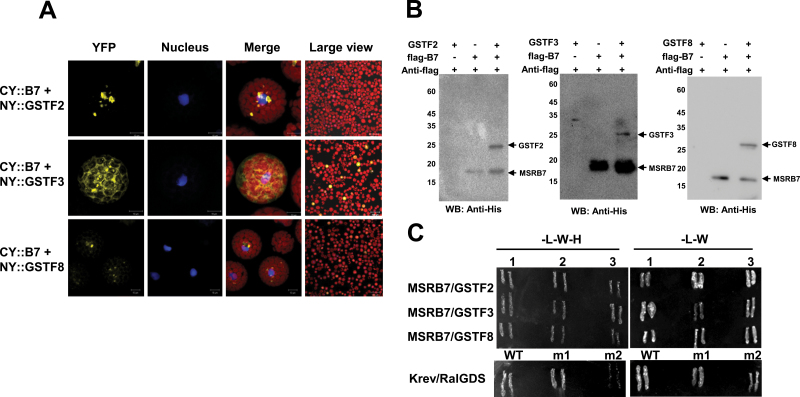
Interaction of MSRB7 with GSTs. (A) BiFC assay for interaction of MSRB7 with GSTs. Yellow indicates CY-B7 [MSRB7 fused with the C-terminal fragment of yellow fluorescence protein (YFP)] and NY-GSTF2/3/8 (GSTF2/3/8 fused with the N-terminal fragment of YFP) dimerization, as determined by BiFC. Red denotes chloroplast autofluorescence and blue denotes nuclear localizing marker (bZIP63–CFP). (B) Co-immunoprecipitation of MSRB7, GSTF2, GSTF3, and GSTF8. Recombinant GSTs proteins were co-precipitated with flag–MSRB7 using an anti-Flag antibody and detected with an anti-His antibody. WB, Western blot. (C) Yeast two-hybrid assay verifying the interactions of GSTF2, GSTF3, and GSTF8 with MSRB7. Controls were performed by co-transforming pEXP32/Krev1 with pEXP22/ RalGDs-WT (strong interaction), pEXP22/RalGDs-m1 (weak interaction), and pEXP22/RalGDs-m2 (no interaction). L, Leu; W, Trp; H, His.

To verify whether the candidate proteins described above interacted with MSRB7, BiFC, co-immunoprecipitation, and yeast two-hybrid assays were performed. A protoplast transient assay revealed the presence of GSTF2, GSTF3, GSTF8, and MSRB7 in the cytosol (Supplementary Fig. S5 available at *JXB* online). GSTF8 has previously been reported to be localized in both the cytosol and plastids (Thatcher *et al.*, 2007), and in our study where the nuclear marker (bZIP63–CFP)-expressing plasmid and BiFC plasmids were co-transformed into *A. thaliana* protoplasts, we observed that GSTF2 and GSTF3 interacted with MSRB7 in the cytosol, while GSTF8 interacted with MSRB7 in close proximity to the chloroplast (Fig. 4A and Supplementary Fig. S6 available at *JXB* online). In addition, we observed that the three GSTF proteins co-immunoprecipitated with MSRB7 and were detected using an anti-His antibody (Fig. 4B). Finally, when MSRB7 was used as the bait protein in the yeast two-hybrid assay, weak interactions with GSTF2, GSTF3, and GSTF8 were observed (Fig. 4C), supporting our observations from the BiFC and co-immunoprecipitation experiments. Together, these results indicated that GSTF2, GSTF3, and GSTF8 are likely to be substrates of MSRB7.

### MSRB7 can restore the activities of oxidized GSTF2 and GSTF3 *in vitro*

**Fig. 5.**
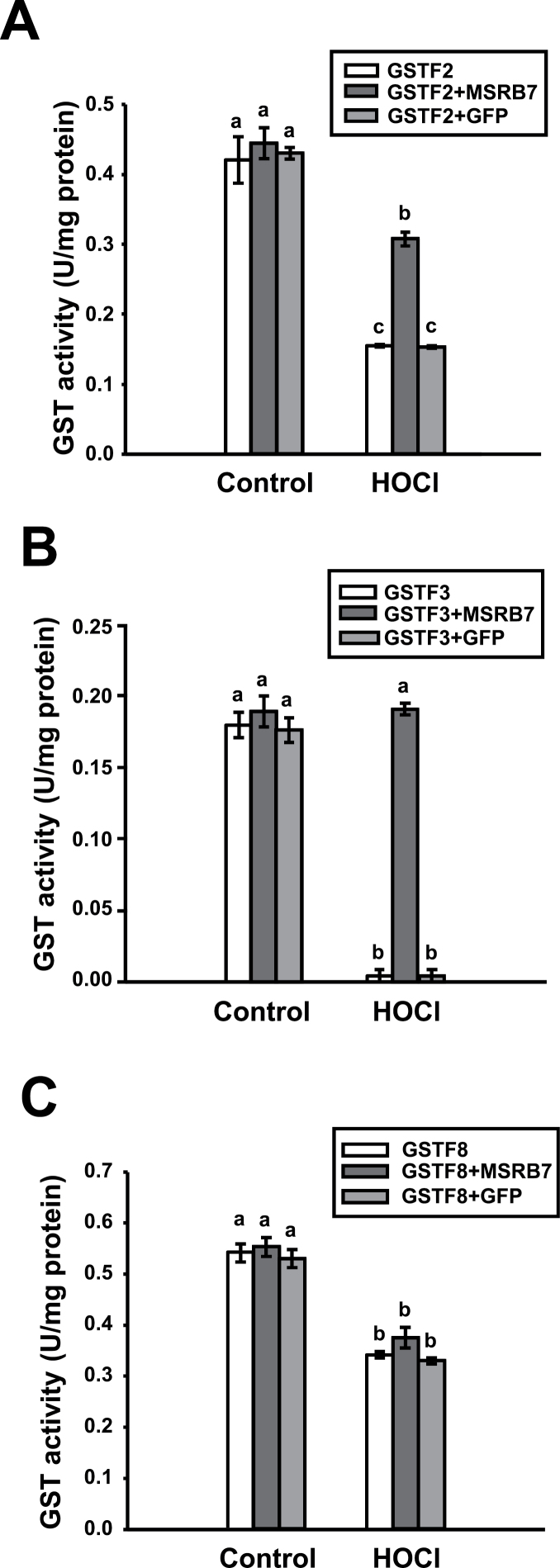
Restoration of oxidized-GST enzymatic activity by MSRB7 *in vitro*. The enzymatic activities of GSTF2 (A), GSTF3 (B), and GSTF8 (C). HOCl-treated recombinant GSTF2, GSTF3, and GSTF8 proteins were co-incubated with MSRB7 for 1h at 25 °C. Enzymatic activities were determined. Recombinant GFP protein was used as a negative control. Data are means±SD (*n*=3) of three independent experiments. Data were analysed statistically using Duncan’s test and different letters indicate significant differences at *P<*0.05.

HOCl is an oxidant that preferentially targets Met residues in proteins, and it has been shown that proteins that are susceptible to oxidative damage can undergo HOCl-mediated oxidation of Met to different extents and lose their functions (Khor *et al.*, 2004). Recombinant GSTF2, GSTF3, GSTF8, and MSRB7 proteins, as well as green fluorescent protein (GFP), were expressed in *E. coli* and purified by Ni^2+^-affinity chromatography. The GST proteins were pre-treated with HOCl to abolish their enzymatic activities, followed by treatment with 5mM Met to terminate the oxidative reaction. To determine whether MSRB7 can restore the enzymatic activities of the MetO–GSTF proteins, HOCl-oxidized or untreated GSTs were co-incubated with MSRB7 or GFP, and the enzymatic activities of the GSTFs were assayed. As expected, HOCl treatment compromised the GST activities (Fig. 5), suggesting that the oxidative damage resulted in loss of protein function. However, when the oxidized proteins were treated with MSRB7, the activities of GSTF2 and GSTF3 were restored to 71 and 100%, respectively (Fig. 5A, B), while no such affect occurred upon treatment with GFP protein, suggesting that the enzymes were repaired specifically by MSRB7 (Fig. 5A, B). Although GSTF8 interacts with MSRB7 (Fig. 4), MetO–GSTF8 activity was not restored upon treatment with MSRB7 (Fig. 5C).

### MSRB7 maintains the stability of GSTF2 and GSTF3 *in vivo*

**Fig. 6.**
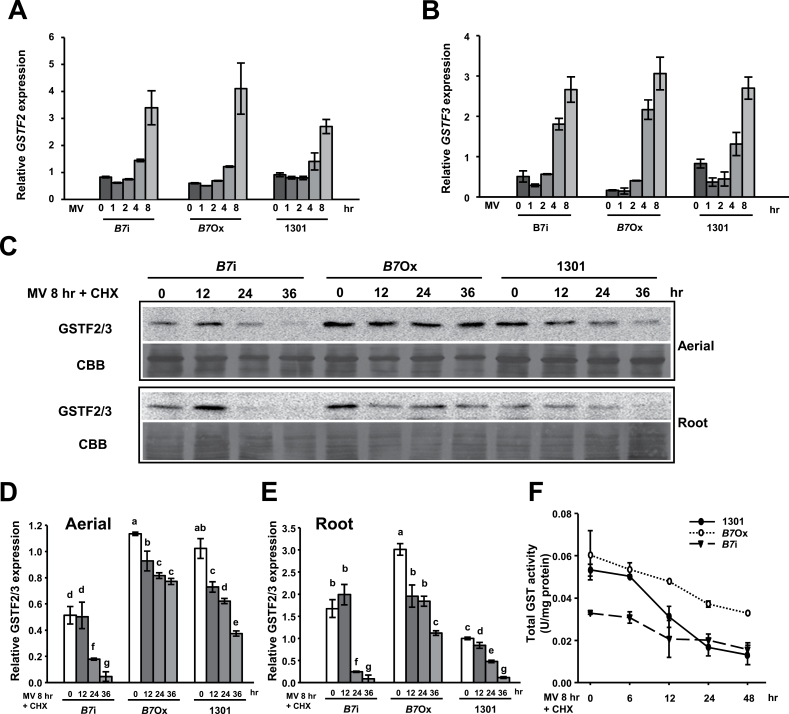
Maintenance of GST stability by MSRB7 *in vivo*. (A, B) Expression patterns of *GSTF2* and *GSTF3*. Real-time PCR analysis of *GSTF2* and *GSTF3* transcripts in 10-d-old *B7*Ox, *B7*i, and 1301 plants treated with 10 μM MV for 8h. (C) Immunoblotting of GSTF2/3 in aerial parts and roots. Ten-day-old 1301, *B7*Ox, and *B7*i seedlings were pre-treated with 10 μM MV for 8h, followed by treatment with 0.5mM CHX for 0–36h. GSTF2/3 was detected using an anti-GSTF2/3 antibody. Protein stained with Coomassie Brilliant Blue (CBB) was used as a protein loading control. (D, E) Relative expression of GSTF2/3. The relative amounts of GSTF2/3 in the aerial parts and roots were determined using immunoblot analysis, and quantified using G:Box iChemi XL (Syngene). Data were analysed statistically using Duncan’s test and different letters indicate significant differences at *P<*0.05. (F) Total GST activity of *MSRB7* transgenic plants. Ten-day-old 1301, *B7*Ox, and *B7*i seedlings were pre-treated with 10 μM MV for 8h followed by treatment with 0.5mM CHX for 0–48h. GST activity was measured. Data represent the means±SD (*n*=10) of three independent experiments.

Since the transcript levels of *GSTF2* and *GSTF3* in wild-type *A. thaliana* are upregulated by oxidative stress (Fig. 3), we evaluated their expression in 1301 (vector-only control), *B7*Ox, and *B7*i plants upon MV treatment. *GSTF2* and *GSTF3* expression was induced by MV treatment (Fig. 6A, B); however, there were no significant differences in the transcript levels in the 1301, *B7*Ox, and *B7*i plants, suggesting that the expression of *GSTF2* and *GSTF3* was not affected by MSRB7. Given that GSTF2 and GSTF3 can interact with MSRB7 (Fig. 4), we examined whether MSRB7 affected the stability of these proteins. Ten-day-old *B7*Ox, *B7*i, and 1301 seedlings were first treated with or without 10 μM MV for 8h to induce GSTF2/3 expression followed by treatment with CHX, an inhibitor of *de novo* protein synthesis, for 0, 12, 24, and 36h. Immunoblot analysis revealed that the endogenous GSTF2/3 protein levels were steadily maintained throughout the 24h post-CHX treatment in 1301, *B7*Ox, and *B7*i plants under normal conditions (Supplementary Fig. S7 available at *JXB* online). However, when the plants were treated with MV, GSTF2/3 protein abundance decreased substantially in both the aerial parts and the roots of *B7*i plants and, importantly, GSTF2/3 protein levels were more stable in both the aerial parts and the roots of *B7Ox* plants than in control 1301 plants (Fig. 6C). Quantitative analysis further showed that GSTF2/3 protein levels were markedly more stable in *B7*Ox than in 1301 and *B7i* plants under oxidative stress (Fig. 6D, E), suggesting that MSRB7 plays a role in maintaining GSTF2 and/or GSTF3 protein stability *in vivo* upon oxidative stress.

Prior to CHX treatment, GST activity was substantially lower in *B7*i plants than in 1301 plants under oxidative stress, but there was no significant difference in GST activity between 1301 and *B7*Ox plants (Fig. 6F). After CHX treatment, the *B7*Ox plants exhibited higher GST activity than the 1301 and *B7*i plants, while GST activity in the 1301 plants decreased rapidly. The activities remained lower in *B7*i than in 1301 and *B7*Ox plants (Fig. 6F). After CHX treatment for 24h, we observed no significant difference in GST activity between the 1301 and *B7*i plants (Fig. 6F). Together, these results suggest that GSTF2/3 activity in plants is maintained and stabilized by MSRB7 under oxidative stress.

### Met residues of GSTF2 and GSTF3 are important for maintaining GST activity, and their oxidized states are reduced by MSRB7

**Fig. 7.**
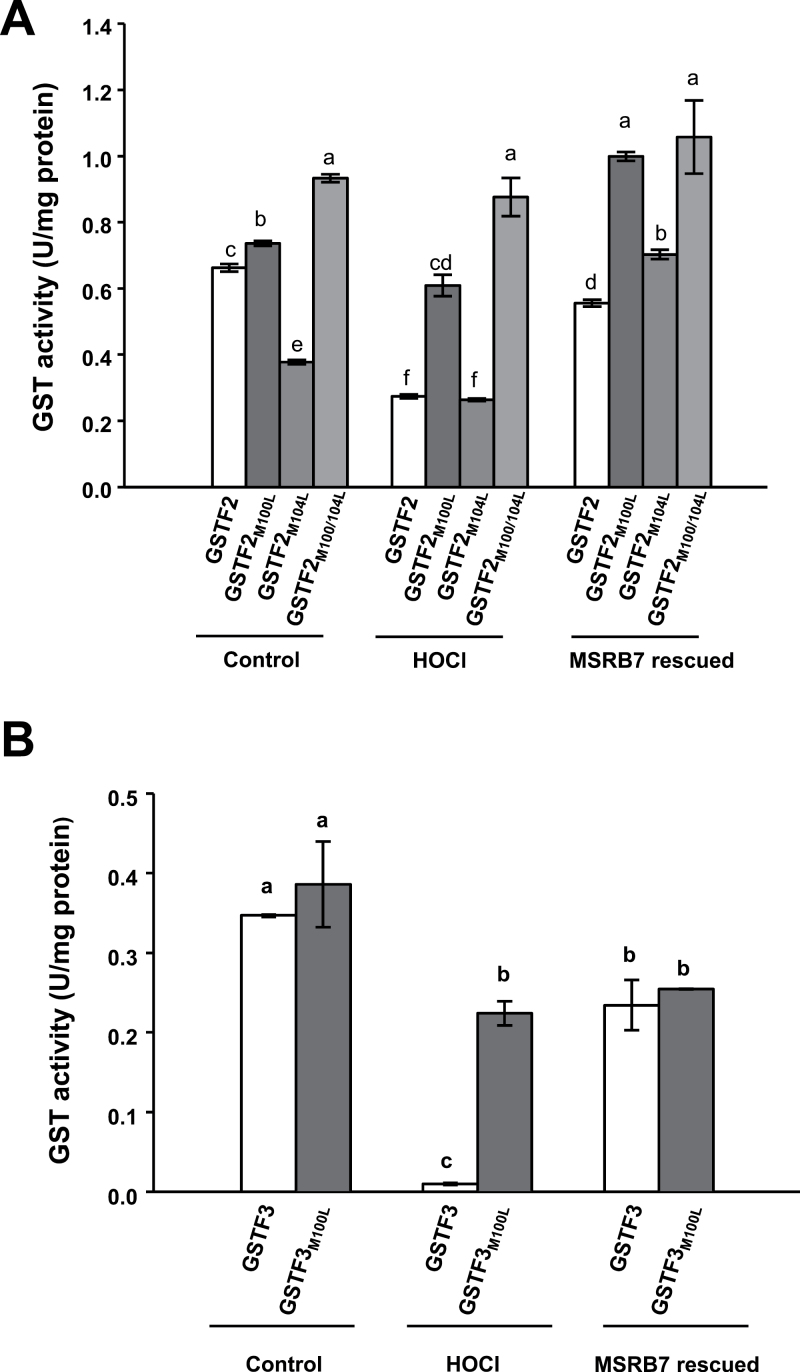
Reduction of Met residues of GSTF2 and GSTF3 by MSRB7. The activities of wild-type and mutated GSTs are shown. HOCl-oxidized recombinant GSTF2, GSTF2_M100L_ (Met replaced with Leu), GSTF2_M104L_, and GSTF2_M100/104L_ (A), and GSTF3 and GSTF3_M100L_ (B) proteins were co-incubated with MSRB7 for 1h at 25 °C and assayed for GST activity. Data denote means±SD (*n*=3) of three independent experiments. Data were analysed statistically using Duncan’s test and different letters indicate significant differences at *P<*0.05.

Since oxidation of GSTF2 and GSTF3 proteins affects their enzymatic activities, we examined the importance of different constituent Met residues under oxidative stress. To this end, recombinant proteins of wild-type GSTF2 and GSTF3, and mutants in which Met residues were replaced with another non-polar amino acid, Leu (GSTF2_M100L_, GSTF2_M104L_, GSTF2_M100/104L_, and GSTF3_M100L_), were generated and purified. Under normal control conditions, the enzymatic activities of all the mutant proteins, except for GSTF2_M104L_, were similar to those of the wild-type GSTF2/3 (Fig. 7). This may suggest that oxidation or reduction of GSTF2_M104_ affects the protein conformation, thereby influencing its enzymatic activity. Upon treatment with HOCl, the enzymatic activities of GSTF2 and GSTF3 were reduced to 42 and 3%, respectively, but were restored to 84 and 70%, respectively, after co-incubation with MSRB7 (Fig. 7). The activities of GSTF2_M100L_, GSTF2_M104L_, and GSTF2_M100/104L_ proteins were slightly affected by the HOCl treatment, and co-incubation with MSRB7 resulted in slight increases in the activities of GSTF2_M100L_ and GSTF2_M104L_ (Fig. 7A). Since the enzymatic activities of these mutants lacking either one or both Met residues were only slightly compromised by the HOCl treatment, it can be concluded that these Met residues are important substrates for oxidative stress and that their oxidized state affects the enzymatic function of GSTF2.

## Discussion

### MSRB7 participates in tolerance to chemically induced ROS

Abiotic stress can induce the accumulation of excess ROS, which are known to damage biomolecules such as Met residues in proteins (Moller and Sweetlove, 2010; Toda *et al.*, 2010). Recent studies have shown that MSRB proteins in *A. thaliana*, rice, and pepper plants have functions related to oxidative stress or defence responses (Vieira Dos Santos *et al.*, 2005; Kwon *et al.*, 2007; Laugier *et al.*, 2010; Oh *et al.*, 2010), and the presence of large numbers of *MSRB* genes suggests that they may have multiple biological functions and protect plants against different types of stress conditions. Overexpression of *MSRB* genes can enhance tolerance to MV and act as non-antibiotic selectable marker genes (Li *et al.*, 2013). This correlates well with the finding that, in *A. thaliana* and tomato plants overexpressing *MSRB7*, there is a decrease in H_2_O_2_ accumulation (Supplementary Fig. S8 available at *JXB* online) accompanied by an increase in GST activity and tolerance to oxidative stress (Li *et al.*, 2012). However, the mechanism by which MSRB7 protects plants against chemically induced oxidative stress remains unclear. In this study, we focused on investigating how oxidized GSTs generated during oxidative stress are reduced by the cytosolic MSRB7 protein and examined the implications of such a mechanism in oxidative stress tolerance.

Based on our proteomic analysis, *B7*ox and *B7*i plants have higher and lower ratios of MetO residues, respectively, in their GSTF2 and GSTF3 proteins (Table 2). This suggests that MSRB7 can protect these proteins against Met oxidation, or that it can convert MetO back to Met following oxidative stress. During the reduction of MetO–GSTs, MSRB7 is itself oxidized, and oxidized proteins are usually unstable and likely to be degraded unless they are reduced by reductases (Kurepa and Smalle, 2008). However, since it has been shown that thioredoxins or glutaredoxin can reduce oxidized MSR proteins via a MSR redox cycle (Tarrago *et al.*, 2009*b*), it is possible that the oxidized MSRB7 is restored to its functional state through this cycle. We observed that MSRB7 protein abundance in the aerial parts was gradually decreased after 4h of MV treatment (Fig. 2D). We propose that this decrease in MSRB7 protein abundance is due to: (i) an inability of the MSR redox cycle to maintain the reduced state of the large amount of oxidized MSRB7 protein, causing it to be degraded; or (ii) adaptation of the plants to oxidative stress such that MSRB7 expression is no longer required. The gradual increase in *MSRB7* mRNA expression in the aerial part in response to MV treatment (Fig. 2A) may be a compensatory response to supplement the degraded MSRB7 protein. Since the level of GSTF2 and GSTF3 expression in the aerial parts of the plant was slightly increased at 4h after MV treatment and remained high throughout (Fig. 3C), we propose that the plant was under constant oxidative stress and did not adapt to such stress.

### Comparative proteomic analysis using CNBr digestion as an efficient strategy for the identification of MSRB7 substrates

To date, no substrates of plant cytosolic MSRBs have been identified. Our data suggest that MSRB7 may protect its target protein(s) against Met oxidation, which would explain the low and high percentages of MetO-containing proteins observed in *B7*ox and *B7*i plants, respectively, during oxidative stress (Table 2). Since CNBr does not cleave proteins/peptides containing MetO, these proteins are not identified by LC-MS/MS analysis. In addition, *B7*i plants contain a higher percentage of MetO, and are therefore not suitable for CNBr-digested comparative proteomic analysis. Using the CNBr approach, 41 stress-related proteins were identified as possible substrates of MSRB7 (Table 1) and GO annotation identified some of these putative substrates as ROS-scavenging proteins, which might account for the enhanced tolerance of *B7*ox plants to oxidative stress.

The total GST activity in *B7*Ox plants is significantly higher than in the wild-type and *B7*i plants (Li *et al.*, 2012), and since GSTF2, GSTF3, and GSTF8 interact with MSRB7 (Fig. 4), they may all be important in protecting plants against oxidative stress. CNBr-cleavable GSTF2 and GSTF3 proteins were detected in *B7*Ox plants during oxidative stress (Table 1). This suggests that the MetO–GSTF2 and MetO–GSTF3 proteins generated during oxidative stress are reduced by the high levels of MSRB7 expressed in *B7*Ox plants. Despite the apparent interaction between GSTF8 and MSRB7 (Fig. 4), CNBr-cleavable GSTF8 was not detected in the MV-treated *B7*Ox plants (Table 1), while high levels, comparable to the other plants, of MetO–GSTF8 were observed (Table 2). The inability of MSRB7 to target repair MetO–GSTF8 protein efficiently may account for the failure to restore the GSTF8 enzymatic activity (Fig. 5C). The structure of GSTF8 has not yet been solved, but one possible explanation for our results is that the surface-exposed Met residue in GSTF8 is not involved in enzymatic activity. However, we cannot rule out the possibility that oxidative stress may have caused the oxidation of other amino acid residues that are not affected by MSRB7 action, hence reducing the enzymatic activity of GSTF8. We propose that the CNBr digestion approach is an efficient tool for comparative proteomics allowing for identification of physiological targets of MSRB7. A similar approach may be used to study the remaining 41 potential substrates of MSRB7. In addition, the functions of MSRB proteins present in other species may be similarly elucidated using this approach.

### MSRB7 maintains the activity and stability of the substrates GSTF2 and GSTF3

Although some studies have reported that the substrates of MSRs are Met-rich proteins (Gustavsson *et al.*, 2002; Sundby *et al.*, 2005; Tarrago *et al.*, 2012), it is interesting that the Met contents of GSTF2 (1.4%) and GSTF3 (0.9%) are lower than average for all proteins (1.7%) (Alamuri and Maier, 2006). The three-dimensional structures of GSTF2 and GSTF3 retrieved from the NCBI database (http://www.ncbi.nlm.nih.gov/Structure/index.shtml) revealed that GSTF2_M100/104_ and GSTF3_M100_ are surface-exposed residues (Supplementary Fig. S4). We therefore propose that the Met residues of GSTF2 and GSTF3 are located in the functional domain (the α-helical domain in the F class of GSTs), which is essential for secondary structure folding and protein function (Fig. 7 and Supplementary Fig. S4 available at *JXB* online). Despite the lack of one Met residue, MSRB7 was able to restore the enzymatic activities of GSTF2_M100L_ and GSTF2_M104L_ proteins to a level higher than those of the control group. One explanation for this is that these recombinant proteins generated from *E. coli* underwent some degree of oxidation during protein preparation, so the enzymatic activities of the control proteins were not maximal. In contrast, recombinant GSTF2_M100/104L_ protein lacks two Met residues and is unlikely to contain any MetO, explaining the high enzymatic activities observed in all three groups (Fig. 7A). The GSTF3_M100L_ protein lacking the Met residue is susceptible to oxidative damage despite the absence of MetO, and this damage is not affected by MSRB7 action (Fig. 7B), suggesting that the oxidative stress results in the oxidation of other amino acids that are not modified by MSRB7.

The fifty-four homologue (Ffh) protein, a signal recognition particle protein of *E. coli*, is reported to be a substrate of MSRs (Ezraty *et al.*, 2004). This protein is remarkably unstable in an *E. coli* mutant lacking *msra* and *msrb* (Ezraty *et al.*, 2004), suggesting that MSRs have roles in maintaining the stability of their substrates. It has been reported previously that GSTs themselves can become oxidized, causing them to either lose their antioxidant function, or become partially degraded (Dixon and Edwards, 2010). The Met residues of GSTs could be a critical requirement for enzymatic activity and plant survival under oxidative stress, so their oxidation is possibly reversed by other MSRs. The experiments shown in Fig. 5 show consistent >80% recovery for GST2/3 activities in response to MSRB7 treatment. We therefore postulate that HOCl may possess a preference for Met *R*-oxidation, and the enzymatic activities of these oxidized GSTF2 and GSTF3 proteins are, therefore, readily recovered by MSRB7. Our observations suggest that the Met residues of GSTF2 and GSTF3 are important for maintaining enzymatic activities, and their non-functional oxidized states are probably important targets for protein repair by MSRB7 (Figs 6 and 7). Both *B7*i and 1301 plants have a tendency to form MetO at GSTF2_M104_ and GSTF3_M100_ during oxidative stress *in vivo* (Table 2). We conclude that GSTF2, GSTF3, and MSRB7 are components of an oxidative stress tolerance mechanism that depends on the maintenance of GSTF2 and GSTF3 activity by MSRB7.

This study identified GSTF2 and GSTF3 as substrates of plant cytosolic MSRB during oxidative stress tolerance. However, the mechanism underlying the maintenance of substrate stability remains to be elucidated. Although MSRB8 shares 95% amino acid identity with MSRB7 and is MV inducible (Li *et al.*, 2012), we believe that MSRB8 may act on substrates other than GSTF2 and GSTF3 as it is incapable of compensating for the loss of MSRB7 activity observed in *B7*i plants. Therefore, in the future, it will be also interesting to identify the substrates of MSRB8, and compare and contrast the functional roles of these two highly homologous proteins.

## Supplementary data

Supplementary data are available at *JXB* online.

Supplementary Fig. S1. Constructs used in the BiFC assay.

Supplementary Fig. S2. Induction of the *MSRB7* promoter by oxidative stress in cauline and rosette leaves but not in flowers and siliques.

Supplementary Fig. S3. Determination of the binding specificities of GSTF2/3-specific antibody against rGSTF2 and rGSTF3 recombinant proteins.

Supplementary Fig. S4. Amino acid sequences of GSTs and the three-dimensional structure of GSTF2.

Supplementary Fig. S5. Cytosolic locations of MSRB7, GSTF2, GSTF3, and GSTF8.

Supplementary Fig. S6. Controls of BiFC assays.

Supplementary Fig. S7. Amounts of GSTF2/3 are not significantly different following CHX treatment.

Supplementary Fig. S8. H_2_O_2_ content.

Supplementary Table S1. LC-MS/MS proteomic analysis of WT and *B7*Ox plants.

Supplementary Table S2. Primers used for PCR and real-time PCR.

Supplementary Data

## Abbreviations:

B7i: MSRB7 knockdown plant
B7Ox: MSRB7 overexpressing plant
BiFC: Bimolecular Fluorescence Complementation
CFP: cyan fluorescent protein
CHX: cycloheximide
CNBr: cyanogen bromide
CDNB: 1-chloro-2,4-dinitrobenzene
DTT: dithiothreitol
GFP: green fluorescent protein
GO: Gene Ontology
GST: glutathione transferase
GUS: β-glucuronidase
HOCl: hypochlorous acid
HSP: heat-shock protein
LC-MS/MS: liquid chromatography tandem mass spectrometry
MetO: methionine sulfoxide
Met-*R*-O: Met-*R*-sulfoxide
Met-*S*-O: Met-*S*-sulfoxide
MSR: methionine sulfoxide reductase
MV: methyl viologen
PLGS: ProteinLynx GlobalServer
PMSF: phenylmethylsulfonyl fluoride
ROS: reactive oxygen species
RT-PCR: reverse transcription-PCR
SD: standard deviation.
