# Supplementary material for: MSRB7 reverses oxidation of GSTF2/3 to confer tolerance of Arabidopsis thaliana to oxidative stress
Source: J Exp Bot. 2014 Jun 24;65(17):5049–62. doi: 10.1093/jxb/eru270 (PMC4144780; doi:10.1093/jxb/eru270)
Supplement: Supplementary Data [file supp_65_17_5049__index.html]

MSRB7 reverses oxidation of GSTF2/3 to confer tolerance of Arabidopsis thaliana to oxidative stress — MSRB7 reverses oxidation of GSTF2/3 to confer tolerance of Arabidopsis thaliana to oxidative stress — Supplementary Data 

# MSRB7 reverses oxidation of GSTF2/3 to confer tolerance of *Arabidopsis thaliana* to oxidative stress

## Supplementary Data

Data files

**Files in this Data Supplement:**

- Supplementary Data - Supplementary Data
