# Supplementary material for: MSRB7 reverses oxidation of GSTF2/3 to confer tolerance of Arabidopsis thaliana to oxidative stress
Source: J Exp Bot. 2014 Jun 24;65(17):5049–62. doi: 10.1093/jxb/eru270 (PMC4144780; doi:10.1093/jxb/eru270)
Supplement: Supplementary Data [file supp_eru270_jexbot128298_file001.pdf]

Supplementary Table S1. LC-MS/MS proteomic analysis of WT and B7Ox plants.

Accession	Description	Score	Unique	B7Ox/WT MV 0 h	B7Ox/WT MV 24h
IPI:IPI00536062.1	Glutathione S transferase F8	117.32	B7Ox 0h	B7Ox 0h	N.D.
IPI:IPI00532125.1	F15I1 12 protein	596.26	B7Ox 0h	B7Ox 0h	N.D.
IPI:IPI00518163.1	Ribulose bisphosphate carboxylase oxygenase activase	291.06	B7Ox 0h	B7Ox 0h	N.D.
IPI:IPI00520309.1	RCA RUBISCO ACTIVASE	291.06	B7Ox 0h	B7Ox 0h	N.D.
IPI:IPI00542532.2	MLP like protein 423	245.59	B7Ox 0h	B7Ox 0h	N.D.
IPI:IPI00516423.1	ACP4 ACYL CARRIER PROTEIN 4	193.68	B7Ox 0h	B7Ox 0h	N.D.
IPI:IPI00656779.1	Fructose bisphosphate aldolase	160.18	B7Ox 0h	B7Ox 0h	N.D.
IPI:IPI00518620.2	Peroxidase 32	147.69	B7Ox 0h	B7Ox 0h	N.D.
IPI:IPI00527268.1	Dynamin family protein	137.57	B7Ox 0h	B7Ox 0h	N.D.
IPI:IPI00539116.1	Myrosinase	135.6	B7Ox 0h	B7Ox 0h	N.D.
IPI:IPI00532571.1	tRNA pseudouridine synthase family protein	129.23	B7Ox 0h	B7Ox 0h	N.D.
IPI:IPI00539324.1	Hydroxyethylthiazole kinase family protein	127.98	B7Ox 0h	B7Ox 0h	N.D.
IPI:IPI00524641.1	Triosephosphate isomerase chloroplastic	127.79	B7Ox 0h	B7Ox 0h	N.D.
IPI:IPI00846597.1	TIM TRIOSEPHOSPHATE ISOMERASE	127.79	B7Ox 0h	B7Ox 0h	N.D.
IPI:IPI00543655.1	Isoform 2 of Citrate synthase 4 mitochondrial	127.39	B7Ox 0h	B7Ox 0h	N.D.
IPI:IPI00532971.1	SHM4 SERINE HYDROXYMETHYLTRANSFERASE 4	126.21	B7Ox 0h	B7Ox 0h	N.D.
IPI:IPI00538916.1	Putative uncharacterized protein	125.68	B7Ox 0h	B7Ox 0h	N.D.
IPI:IPI00523903.1	Heat shock cognate 70 kDa protein 2	121.8	B7Ox 0h	B7Ox 0h	N.D.
IPI:IPI00526611.1	ERD2 (HSP70)	116.83	B7Ox 0h	B7Ox 0h	N.D.
IPI:IPI00539598.1	Trehalose 6 phosphate phosphatase	115.52	B7Ox 0h	B7Ox 0h	N.D.
IPI:IPI00547926.1	ACTIN 2	115.01	B7Ox 0h	B7Ox 0h	N.D.
IPI:IPI00547512.1	RPT4A regulatory particle triple A 4A	112.56	B7Ox 0h	B7Ox 0h	N.D.
IPI:IPI00541076.1	F8A5 6 protein	111.12	B7Ox 0h	B7Ox 0h	N.D.
IPI:IPI00538349.1	Monodehydroascorbate reductase	103.82	B7Ox 0h	B7Ox 0h	N.D.
IPI:IPI00531336.1	Similar to RPT4A regulatory particle triple A 4A ATPase	98.27	B7Ox 0h	B7Ox 0h	N.D.
IPI:IPI00540828.1	At1g60530 F8A5 7 : GTP binding, GTPase activity	96.68	B7Ox 0h	B7Ox 0h	N.D.

IPI:IPI00539389.1	HSP70B heat shock protein 70B	96.29	B7Ox 0h	B7Ox 0h	N.D.
IPI:IPI00539339.1	Ribose 5 phosphate isomerase related	91.58	B7Ox 0h	B7Ox 0h	N.D.
IPI:IPI00529374.1	Cysteine synthase chloroplastic chromoplastic	86.77	B7Ox 0h	B7Ox 0h	N.D.
IPI:IPI00516325.1	Isoform 1 of Ubiquitin carboxyl terminal hydrolase 27	86.62	B7Ox 0h	B7Ox 0h	N.D.
IPI:IPI00545934.1	Tubulin beta 6 chain	84.79	B7Ox 0h	B7Ox 0h	N.D.
IPI:IPI00530539.1	DCT DIT2 1 DICARBOXYLATE TRANSPORT	84.74	B7Ox 0h	B7Ox 0h	N.D.
IPI:IPI00542280.2	Similar to unknown protein	83.9	B7Ox 0h	B7Ox 0h	N.D.
IPI:IPI00527222.1	Ubiquinol cytochrome C reductase complex	82.91	B7Ox 0h	B7Ox 0h	N.D.
IPI:IPI00525001.1	Tubulin beta 2 beta 3 chain	82.18	B7Ox 0h	B7Ox 0h	N.D.
IPI:IPI00523675.1	Isoform 2 of Cysteine rich receptor like protein kinase 13	79.72	B7Ox 0h	B7Ox 0h	N.D.
IPI:IPI00518916.1	Soluble starch synthase chloroplastic amyloplastic	78.84	B7Ox 0h	B7Ox 0h	N.D.
IPI:IPI00517585.1	Transducin family protein	76.69	B7Ox 0h	B7Ox 0h	N.D.
IPI:IPI00530974.1	Phototropic responsive NPH3 family protein	76.21	B7Ox 0h	B7Ox 0h	N.D.
IPI:IPI00526496.2	Putative GTP binding protein	76.09	B7Ox 0h	B7Ox 0h	N.D.
IPI:IPI00656706.1	Similar to ribulose bisphosphate carboxylase small chain 2B	2291.48	B7Ox 24h	N.D.	B7Ox 24h
IPI:IPI00538684.1	Unknown protein	351.82	B7Ox 24h	N.D.	B7Ox 24h
IPI:IPI00520226.1	Tubulin alpha 6 chain	261.54	B7Ox 24h	N.D.	B7Ox 24h
IPI:IPI00543066.1	Emsy N terminus domain containing protein	195.4	B7Ox 24h	N.D.	B7Ox 24h
IPI:IPI00544767.1	KH domain containing protein	184.86	B7Ox 24h	N.D.	B7Ox 24h
IPI:IPI00532945.1	Glutathione S transferase F3	170.04	B7Ox 24h	N.D.	B7Ox 24h
IPI:IPI00540665.1	ATIPT4 Arabidopsis thaliana isopentenyltransferase 4	140.84	B7Ox 24h	N.D.	B7Ox 24h
IPI:IPI00532024.1	DJ 1 family protein	135.06	B7Ox 24h	N.D.	B7Ox 24h
IPI:IPI00533771.1	Putative disease resistance protein	109.18	B7Ox 24h	N.D.	B7Ox 24h
IPI:IPI00532726.1	Similar to unknown protein	102.22	B7Ox 24h	N.D.	B7Ox 24h
IPI:IPI00544626.1	Isoform 1 of Carbonic anhydrase chloroplastic	756.83	B7Ox 24h	N.D.	B7Ox 24h
IPI:IPI00530621.1	DHAR1 DEHYDROASCORBATE REDUCTASE	155.99	B7Ox 24h	N.D.	B7Ox 24h
IPI:IPI00537995.1	Annexin D1	218.24	B7Ox only	B7Ox 0h	B7Ox 24h
IPI:IPI00535149.3	Glutathione S transferase F2	188.83	B7Ox only	B7Ox 0h	B7Ox 24h

IPI:IPI00525727.1	Serine hydroxymethyltransferase mitochondrial	78.56	B7Ox only	B7Ox 0h	B7Ox 24h
IPI:IPI00543566.1	Malate dehydrogenase 1 mitochondrial	314.93	WT 24h	N.D.	WT 24h
IPI:IPI00516681.1	60S ribosomal protein L12 1	221.4	WT 24h	N.D.	WT 24h
IPI:IPI00540499.1	60S ribosomal protein L12 3	204.57	WT 24h	N.D.	WT 24h
IPI:IPI00539595.1	60S ribosomal protein L6 2	199.18	WT 24h	N.D.	WT 24h
IPI:IPI00543748.1	60S ribosomal protein L6 3	199.18	WT 24h	N.D.	WT 24h
IPI:IPI00545323.1	Adenosine kinase 1	168.54	WT 24h	N.D.	WT 24h
IPI:IPI00530001.1	14 3 3 like protein GF14 upsilon	163.91	WT 24h	N.D.	WT 24h
IPI:IPI00545903.2	S adenosylmethionine synthetase 1	159.37	WT 24h	N.D.	WT 24h
IPI:IPI00537614.1	GRP2 COLD SHOCK DOMAIN PROTEIN 2	152.97	WT 24h	N.D.	WT 24h
IPI:IPI00542050.1	Tubulin beta 4 chain	151.95	WT 24h	N.D.	WT 24h
IPI:IPI00545413.1	Tubulin beta 7 chain	146.02	WT 24h	N.D.	WT 24h
IPI:IPI00527623.1	EMB1879 EMBRYO DEFECTIVE 1879	144.69	WT 24h	N.D.	WT 24h
IPI:IPI00526310.1	Enolase	144.17	WT 24h	N.D.	WT 24h
IPI:IPI00656640.1	PAD1 20S proteasome alpha subunit D1	141.05	WT 24h	N.D.	WT 24h
IPI:IPI00544641.1	Fructose bisphosphate aldolase	139.12	WT 24h	N.D.	WT 24h
IPI:IPI00520872.3	40S ribosomal protein S3a 1	134.37	WT 24h	N.D.	WT 24h
IPI:IPI00545883.1	Oxygen evolving enhancer protein 1 1 chloroplastic	124.43	WT 24h	N.D.	WT 24h
IPI:IPI00523164.1	Adenosine kinase 2	105.64	WT 24h	N.D.	WT 24h
IPI:IPI00846962.1	Malate dehydrogenase NAD	104.28	WT 24h	N.D.	WT 24h
IPI:IPI00540686.1	Actin 2	101.73	WT 24h	N.D.	WT 24h
IPI:IPI00528538.1	Pectin methylesterase	101.3	WT 24h	N.D.	WT 24h
IPI:IPI00527830.1	Probable UTP glucose 1 phosphate uridylyltransferase 2	99.52	WT 24h	N.D.	WT 24h
IPI:IPI00524872.1	ATOEP16	98.87	WT 24h	N.D.	WT 24h
IPI:IPI00536803.2	Nitrilase 1	87.83	WT 24h	N.D.	WT 24h
IPI:IPI00541694.1	Phosphoenolpyruvate carboxylase 1	80.27	WT 24h	N.D.	WT 24h
IPI:IPI00531212.2	Similar to unknown protein	74.82	WT 24h	N.D.	WT 24h
IPI:IPI00547305.1	UPF0497 membrane protein At5g40300	71.24	WT 24h	N.D.	WT 24h

IPI:IPI00523988.1	F15I1 9 protein	69.65	WT 24h	N.D.	WT 24h
IPI:IPI00527713.1	Adenosylhomocysteinase 1	253.3	WT 0 h	WT 0 h	N.D.
IPI:IPI00528647.1	Elongation factor 1 beta 2	240.67	WT 0 h	WT 0 h	N.D.
IPI:IPI00531081.1	2, 3, bisphosphoglycerate independent phosphoglycerate mutase 1	187.78	WT 0 h	WT 0 h	N.D.
IPI:IPI00523898.1	Nascent polypeptide associated complex subunit alpha like protein 2	167.06	WT 0 h	WT 0 h	N.D.
IPI:IPI00520908.1	Catalase 1	150.69	WT 0 h	WT 0 h	N.D.
IPI:IPI00516781.1	Putative uncharacterized protein T1E4 3	132.41	WT 0 h	WT 0 h	N.D.
IPI:IPI00530493.1	Cold responsive protein	128.61	WT 0 h	WT 0 h	N.D.
IPI:IPI00527089.1	Adenosylhomocysteinase 2	116.32	WT 0 h	WT 0 h	N.D.
IPI:IPI00524611.1	Actin 7	115.07	WT 0 h	WT 0 h	N.D.
IPI:IPI00534593.1	Cell division protein ftsZ homolog chloroplastic	100.32	WT 0 h	WT 0 h	N.D.
IPI:IPI00527972.1	methionine adenosyltransferase 2	93	WT 0 h	WT 0 h	N.D.
IPI:IPI00657575.1	Structural constituent of ribosome	287.72	WT 0h	WT 0h	N.D.
IPI:IPI00547933.1	Tubulin alpha 2 alpha 4 chain	261.54	WT 0h	WT 0h	N.D.
IPI:IPI00846137.1	Elongation factor 1 alpha	236.16	WT 0h	WT 0h	N.D.
IPI:IPI00534852.1	Glutamine synthetase chloroplastic mitochondrial	209.67	WT 0h	WT 0h	N.D.
IPI:IPI00656964.1	ATGHBDH GHBDH	164.6	WT 0h	WT 0h	N.D.
IPI:IPI00536776.1	26S proteasome regulatory subunit	137.82	WT 0h	WT 0h	N.D.
IPI:IPI00852300.1	Putative uncharacterized protein M3E9 150	115.64	WT 0h	WT 0h	N.D.
IPI:IPI00538017.1	Actin 11	115.26	WT 0h	WT 0h	N.D.
IPI:IPI00539026.1	Meprin and TRAF homology domain containing protein	112.55	WT 0h	WT 0h	N.D.
IPI:IPI00544270.1	Similar to unknown protein	110.63	WT 0h	WT 0h	N.D.
IPI:IPI00535813.1	Actin 13	104.28	WT 0h	WT 0h	N.D.
IPI:IPI00543401.1	HHP5 heptahelical protein 5	103.5	WT 0h	WT 0h	N.D.
IPI:IPI00545907.1	Tetrahydrofolate dehydrogenase cyclohydrolase	89.97	WT 0h	WT 0h	N.D.
IPI:IPI00541162.1	UDP 3 0 acyl N acetylglucosamine deacetylase family protein	79.86	WT 0h	WT 0h	N.D.
IPI:IPI00537168.1	Isoform 1 of 60S ribosomal protein L11 2	494.29		B7Ox 0h	B7Ox 24h
IPI:IPI00534991.1	Identical to Phosphoglycerate kinase chloroplast precursor	339.59		B7Ox 0h	B7Ox 24h

IPI:IPI00523477.1	Ribulose bisphosphate carboxylase small chain 2B chloroplastic	2906.54	3.03	B7Ox 24h
IPI:IPI00532772.1	Glutamine synthetase cytosolic isozyme 1	254.75	1.47	B7Ox 24h
IPI:IPI00548101.1	Uncharacterized protein At1g09340 chloroplastic	205.19	0.97	B7Ox 24h
IPI:IPI00891178.1	Similar to glyceraldehyde 3 phosphate	1946.67	0.87	B7Ox 24h
IPI:IPI00523587.1	Carbonic anhydrase 2	355.49	WT 0h	0.52
IPI:IPI00517953.1	60S ribosomal protein L8 1	156.5	B7Ox 0h	0.58
IPI:IPI00521214.1	NDPK1 nucleoside diphosphate kinase 1	130.96	0.86	0.58
IPI:IPI00846186.1	DHAR1 DEHYDROASCORBATE REDUCTASE	155.99	1	0.62
IPI:IPI00522440.1	5 methyltetrahydropteroyltriglutamate homocysteine methyltransferase	526.63	0.85	0.66
IPI:IPI00533310.1	40S ribosomal protein S7 1	363.05	B7Ox 0h	0.67
IPI:IPI00518464.1	Isoform 3 of Carbonic anhydrase chloroplastic	926.79	2.38	0.67
IPI:IPI00543463.1	Malate dehydrogenase cytoplasmic 2	143.51	B7Ox 0h	0.68
IPI:IPI00518864.1	Malate dehydrogenase cytoplasmic 1	235.66	1.09	0.74
IPI:IPI00532582.1	Isoform 2 of Oxygen evolving enhancer protein 3	296.93	B7Ox 0h	0.75
IPI:IPI00656928.1	CATALASE 2	108.27	1.54	0.76
IPI:IPI00517335.1	Elongation factor EF 2	905.32	0.8	0.77
IPI:IPI00519410.1	Chaperonin	155.36	WT 0h	0.78
IPI:IPI00525237.1	RuBisCO large subunit binding protein subunit alpha chloroplastic	364.75	0.66	0.79
IPI:IPI00537354.1	20 kDa chaperonin chloroplastic	238.12	0.74	0.79
IPI:IPI00544292.1	RuBisCO large subunit binding protein subunit beta chloroplastic	200.59	0.66	0.85
IPI:IPI00846291.1	HOG1 HOMOLOGY DEPENDENT GENE SILENCING 1	108.26	1	0.88
IPI:IPI00538665.1	PGK PHOSPHOGLYCERATE KINASE	191.52	B7Ox 0h	0.98
IPI:IPI00531983.1	Major latex protein related	900.21	WT 0h	0.99
IPI:IPI00537782.1	Transketolase like protein	443.73	1.06	1.02
IPI:IPI00532889.1	Encodes a cytosolic methionine synthase	198.16	1.02	1.05
IPI:IPI00535114.1	Ribulose bisphosphate carboxylase large chain	570.44	1.64	1.10
IPI:IPI00542693.1	TUA6 tubulin alpha 6 chiain	261.54	0.99	1.12
IPI:IPI00545353.1	Ribulose bisphosphate carboxylase small chain 3B chloroplastic	2906.54	B7Ox 0h	1.15

IPI:IPI00529143.1	Tubulin alpha 3 alpha 5 chain	266.79	0.97	1.15
IPI:IPI00539020.1	Ribulose bisphosphate carboxylase small chain 1A chloroplastic	3213.28	1.59	1.16
IPI:IPI00541448.2	Probable fructose bisphosphate aldolase 2 chloroplastic	898.54	0.98	1.19
IPI:IPI00521186.1	Ribulose bisphosphate carboxylase small chain 1B chloroplastic	2947.88	2.13	1.20
IPI:IPI00535490.1	PGK1 PHOSPHOGLYCERATE KINASE 1	475.57	1.09	1.28
IPI:IPI00537112.1	Heat shock protein 81 2	203.99	1	1.28
IPI:IPI00530695.1	Phosphoglycerate kinase chloroplastic	456.51	1.06	1.32
IPI:IPI00537303.1	Glyceraldehyde 3 phosphate dehydrogenase A chloroplastic	2113.34	0.94	1.32
IPI:IPI00520474.1	Elongation factor Tu chloroplastic	225.7	0.94	1.32
IPI:IPI00544582.1	Similar to glyceraldehyde 3 phosphate	1946.67	20	1.33
IPI:IPI00548616.1	Oxygen evolving enhancer protein 3 2 chloroplastic	289.21	1.37	1.37
IPI:IPI00548920.2	ATP dependent Clp protease proteolytic subunit 4 chloroplastic	525.04	0.77	1.37
IPI:IPI00544876.1	Sedoheptulose 1 7 bisphosphatase chloroplastic	111.61	B7Ox 0h	1.41
IPI:IPI00533497.1	Beta glucosidase	102.19	B7Ox 0h	1.43
IPI:IPI00541680.1	Glyceraldehyde 3 phosphate dehydrogenase B chloroplastic	634.55	1.22	1.72
IPI:IPI00534087.1	Heat shock protein 81 3	203.99	B7Ox 0h	2.38
IPI:IPI00531652.1	Malate dehydrogenase	149.17	B7Ox 0h	2.44
IPI:IPI00657124.1	Isoform 1 of Oxygen evolving enhancer protein 3 1 chloroplastic	296.93	WT 0h	WT 24h
IPI:IPI00518635.1	F18O14 33	155.99	WT 0h	WT 24h
IPI:IPI00536966.1	MTO3 S adenosylmethionine synthase 3	154.84	WT 0h	WT 24h
IPI:IPI00543293.1	Heat shock cognate 70 kDa protein 1	118.63	WT 0h	WT 24h
IPI:IPI00890975.1	Identical to Phosphoglycerate kinase chloroplast precursor	336.67	B7Ox 0h	WT 24h
IPI:IPI00517861.1	Fructose bisphosphate aldolase	225.43	B7Ox 0h	WT 24h
IPI:IPI00540396.1	Serine glyoxylate aminotransferase	215.13	B7Ox 0h	WT 24h
IPI:IPI00549121.1	Triosephosphate isomerase cytosolic	204.61	B7Ox 0h	WT 24h
IPI:IPI00524343.1	Malate dehydrogenase chloroplastic	121.7	B7Ox 0h	WT 24h
IPI:IPI00891079.1	Encodes a member of heat shock protein 70 family	118.63	B7Ox 0h	WT 24h
IPI:IPI00541659.1	AT3g18780 MVE11 16	101.95	B7Ox 0h	WT 24h

IPI:IPI00524040.1	Actin 8	101.73	B7Ox 0h	WT 24h
IPI:IPI00530401.3	Phosphoenolpyruvate carboxylase 2	98.75	B7Ox 0h	WT 24h
IPI:IPI00519778.1	Pyruvate kinase	97.1	B7Ox 0h	WT 24h
IPI:IPI00522131.1	Phosphoenolpyruvate carboxylase 3	91.32	B7Ox 0h	WT 24h
IPI:IPI00536892.1	Similar to fructose bisphosphate aldolase	93.95	1.01	WT 24h
IPI:IPI00538278.1	Aminomethyltransferase mitochondrial	183.6	0.97	WT 24h
IPI:IPI00846497.1	GAPC 2	165.35	0.92	WT 24h
IPI:IPI00544533.1	Ribosomal protein L9 5 partial Fragment	508.87	0.86	WT 24h
IPI:IPI00522202.1	60S ribosomal protein L6 1	185.71	0.75	WT 24h
IPI:IPI00524577.1	40S ribosomal protein S8 1	210.9	0.68	WT 24h
IPI:IPI00533295.2	14 3 3 like protein GF14 chi	174.48	0.68	WT 24h
IPI:IPI00539263.1	2 cys peroxiredoxin	111.46	1	WT 24h
IPI:IPI00517188.1	CA2 BETA CARBONIC ANHYDRASE 2	356.42	B7Ox 0h	WT 24h
IPI:IPI00542524.1	Dehydrin ERD14	302.6	WT 0h	1
IPI:IPI00891841.1	Similar to ribulose bisphosphate carboxylase small chain 2B	2291.48	B7Ox 0h	1
IPI:IPI00846574.1	CA2 BETA CARBONIC ANHYDRASE 2	330.81	B7Ox 0h	1
IPI:IPI00518090.1	GAPC 2	190.2	B7Ox 0h	1
IPI:IPI00536510.1	Isoform 2 of Carbonic anhydrase chloroplastic	756.83	2.78	1
IPI:IPI00540922.1	Heat shock protein 81 4 HSP81 4	213.67	0.86	1
IPI:IPI00526535.1	2 Cys peroxiredoxin BAS1 chloroplastic	133.42	0.82	1
IPI:IPI00519748.1	Glutamine synthetase cytosolic isozyme 1 1	256.82	0.74	1
IPI:IPI00526733.1	Ribulose bisphosphate carboxylase oxygenase activase	291.06	0.72	1

N.D. means not determined.

A

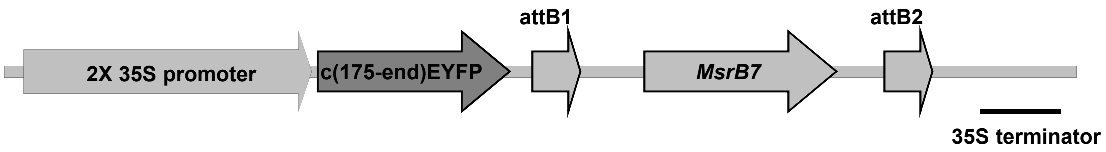

B

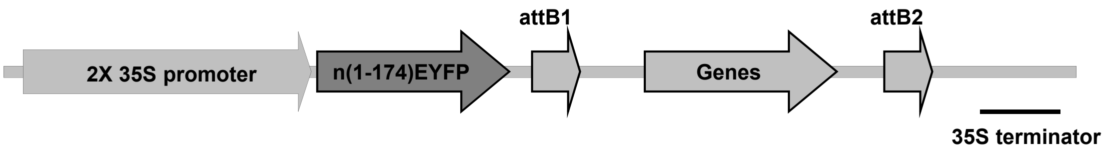

Supplementary Fig. S1. Constructs used in the BiFC assay.
(A, B), constructs of cY::B7 [(C terminal YFP (yellow fluorescent protein)] was fused with MSRB7) and nY::candidate genes (N terminal YFP was fused with candidate genes).

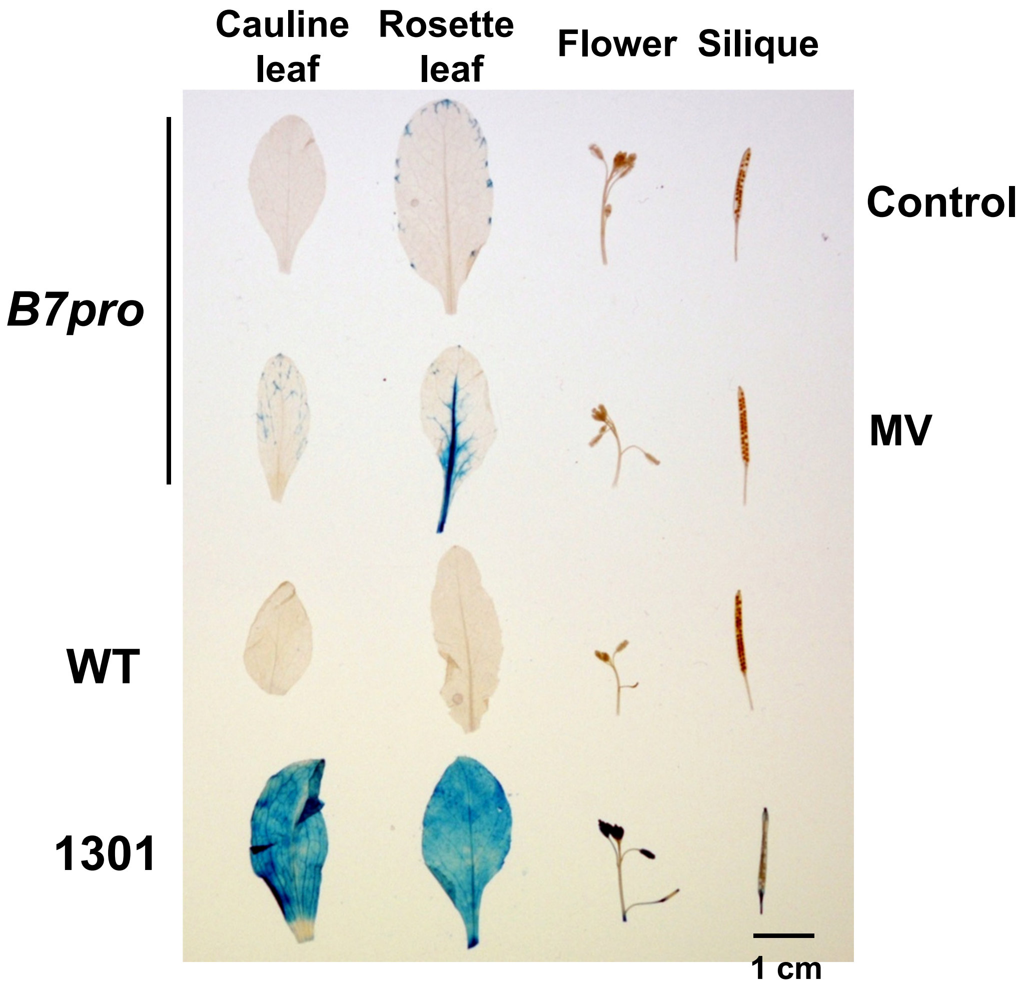

Supplementary Fig. S2. Induction of the *MSRB7* promoter by oxidative stress in cauline and rosette leaves but not in flowers and siliques.
Histochemical GUS staining. Six-week old Arabidopsis seedlings with *MSRB7* promoter (*B7pro*)-driven *GUS* expression were treated with or without 10 μM MV for 8 h and GUS staining was performed. Wild type (WT) and pCAMBIA1301 transgenic plants (*CaMV35Spro*-GUS; 1301) were used as negative and positive controls, respectively.

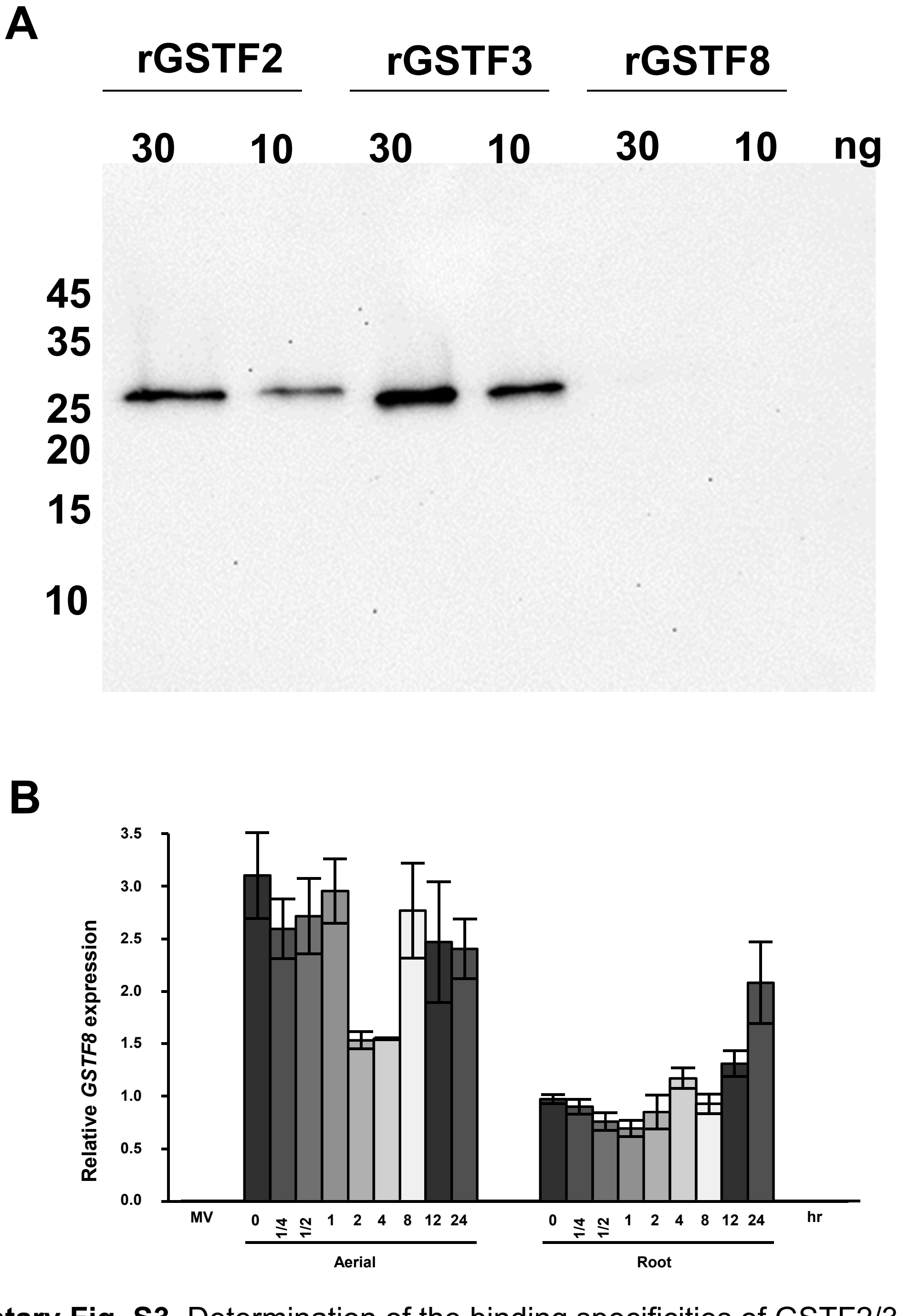

Supplementary Fig. S3. Determination of the binding specificities of GSTF2/3-specific antibody against rGSTF2 and rGSTF3 recombinant proteins. *GSTF8* is not MV-inducible. (A), the GSTF2/3 antibody recognized both the recombinant proteins rGSTF2 and rGSTF3, but not rGSTF8 *in vitro*. (B), *GSTF8* was not MV-inducible. Real-time PCR analysis of transcripts in 10-day-old Arabidopsis plants treated with 10 μM MV for 15 min to 24 h. The relative expression patterns were calculated using *EF1α* and *18S* rRNA as internal references. Data are means ± SD (n = 10) of three independent experiments.

A

The amino acids of GSTF2

1	MAGIKVFGHP	ASIATRRVLI	ALHEKNLDFE	LVHVELKDGE	HKKEPFLSRN
51	PFGQVPAFED	GDLKLFESRA	ITQYIAHRYE	NQGTNLLQTD	SKNISQYAIM
101	AIGMQVEDHQ	FDPVASKLAF	EQIFKSIYGL	TTDEAVVAEE	EAKLAKVLDV
151	YEARLKEFKY	LAGETFTLTD	LHHIPAIQYL	LGTPTKKLFT	ERPRVNEWVA
201	EITKRPASEK	VQ			

B

The amino acids of GSTF3

1	MAGIKVFGHP	ASTSTRRVLI	ALHEKNLDFE	LVHVELKDGE	HKKEPFLSRN
51	PFGQVPAFED	GDLKLFESRA	ITQYIAHRYE	NQGTNLLPAD	SKNIAQYAIM
101	SIGIQVEAHQ	FDPVASKLAW	EQVFKFNYGL	NTDQAVVAEE	EAKLAKVLDV
151	YEARLKEFKY	LAGETFTLTD	LHHIPVIQYL	LGTPTKKLFT	ERPRVNEWVA
201	EITKRPASEK	VL			

C

The amino acids of GSTF8

1	MGAIQARLPL	FLSPPSIKHH	TFLHSSSSNS	NFKIRSNKSS	SSSSSSIIMA
51	SIKVHGVPMS	TATMRVLATL	YEKDLQFELI	PVDMRAGAHK	QEAHLALNPF
101	GQIPALEDGD	LTLFESRAIT	QYLAEEYSEK	GEKLISQDCK	KVKATTNVWL
151	QVEGQQFDPN	ASKLAFERVF	KGMFGMTTDP	AAVQELEGKL	QKVLDVYEAR
201	LAKSEFLAGD	SFTLADLHHL	PAIHYLLGTD	SKVLFDSRPK	VSEWIKKISA
251	RPAWAKVIDL	QKQ			

D

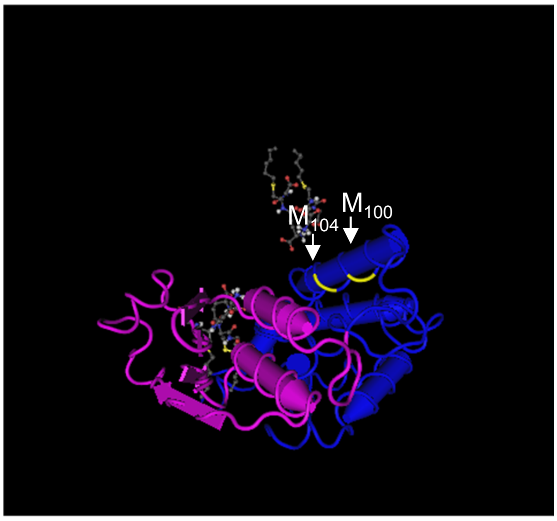

Supplementary Fig. S4. Amino acid sequences of GSTs and the 3-D structure of GSTF2. (A), the amino acid sequence of GSTF2. (B), the amino acid sequence of GSTF3. (C), the amino acid sequence of GSTF8. (D), the 3-D structure of GSTF2 retrieved from the NCBI database (http://www.ncbi.nlm.nih.gov/Structure/index.shtml) reveals that $GSTF2_{M100/104}$ are surface-exposed residues.

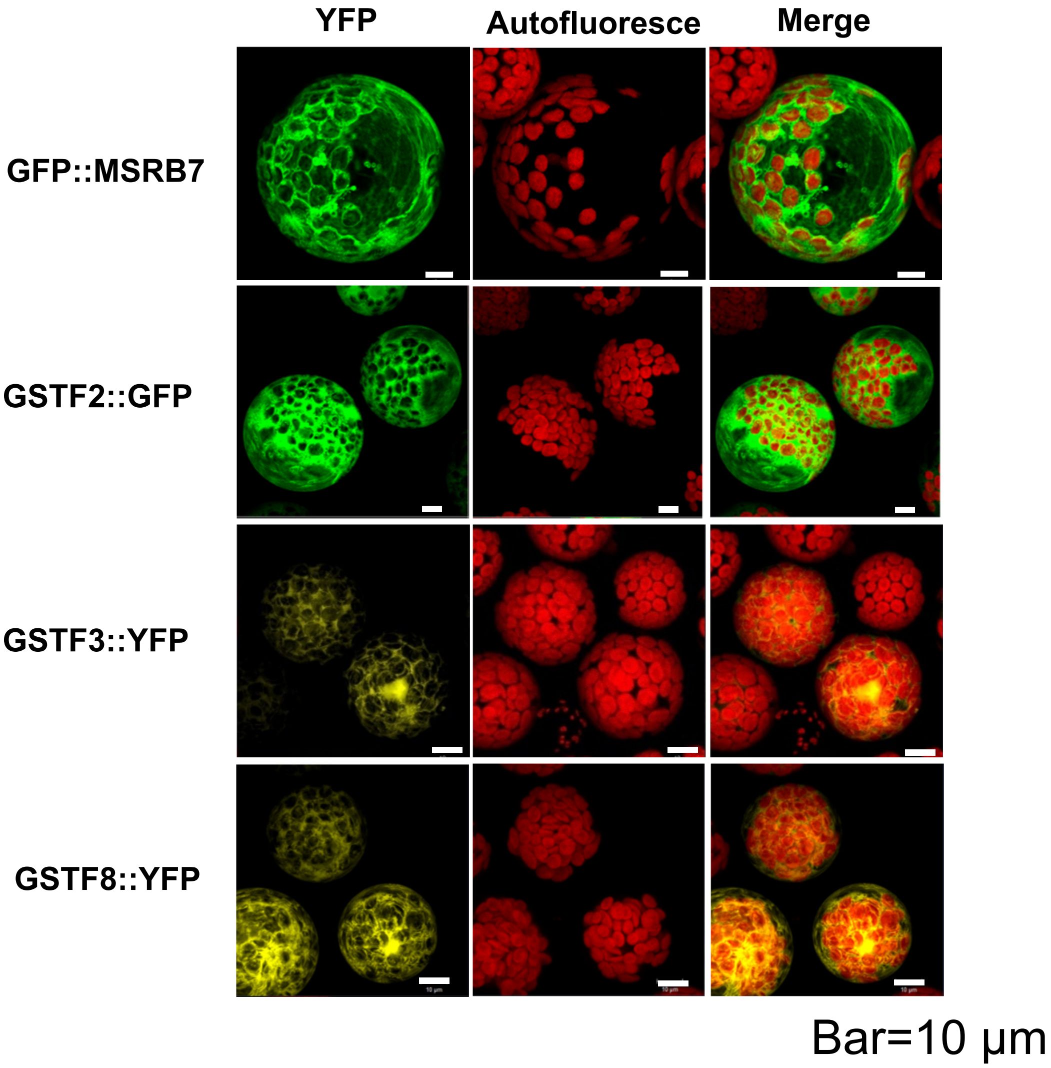

Supplementary Fig. S5. Cytosolic locations of MSRB7, GSTF2, GSTF3, and GSTF8. MSRB7 N-terminus was fused with GFP (GFP::MSRB7). GSTF2 C-terminal was fused with GFP (GSTF2::GFP). GSTF3 and GSTF8 C-terminals were fused with YFP (GST::YFP). Proteins were localized using a Zeiss LSM510 META laser scanning confocal microscope.

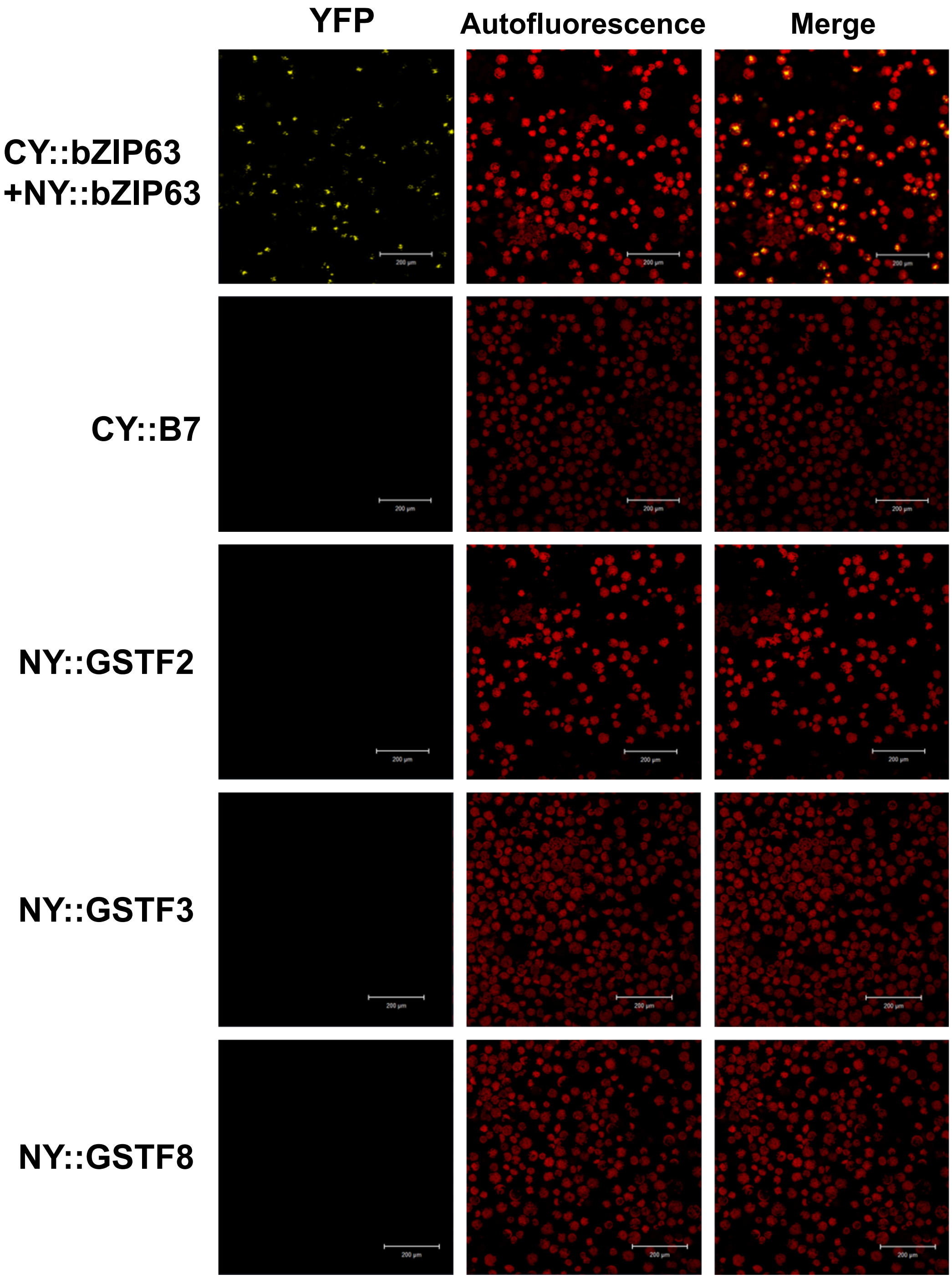

Supplementary Fig. S6. Controls of BiFC assays.
Yellow color indicates CY-bZIP63 (bZIP63 fused with cYFP) and NY-bZIP63 (bZIP63 fused with nYFP) homo-dimerization (Wu *et al.*, 2009), as determined by BiFC. CY-B7 (MSRB7 fused with cYFP) and NY-GSTF2/3/8 (GSTF2/3/8 fused with nYFP); Red, chloroplast auto-fluorescence.

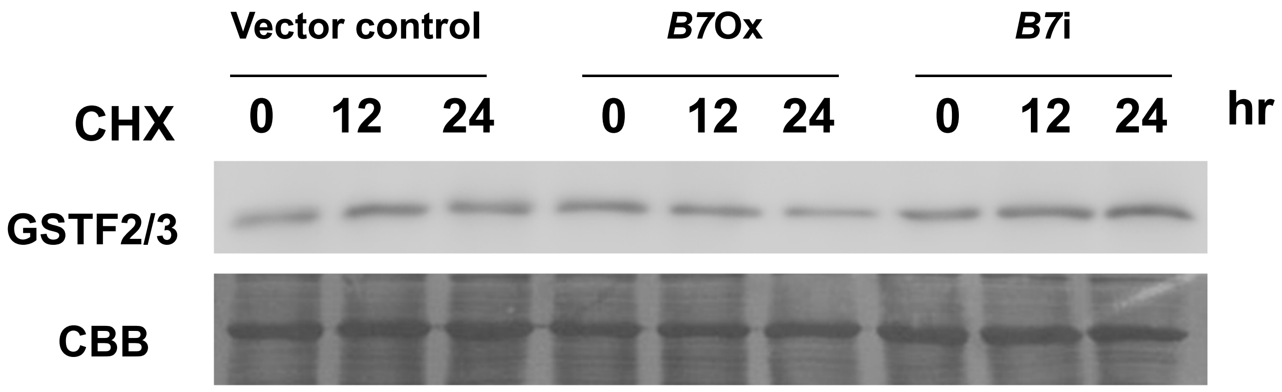

Supplementary Fig. S7. Amounts of GSTF2/3 were not significantly different following CHX treatment.

The amounts of GSTF2/3 in aerial and root parts were detected by immunoblotting. Ten-day-old 1301, *B7*Ox, and *B7*i seedlings were treated with 0.5 mM cycloheximide for 0 to 24 h. GSTF2/3 was detected using a GSTF2/3-specific antibody. Protein stained with Coomassie Brilliant Blue (CBB) was used as a protein loading control.

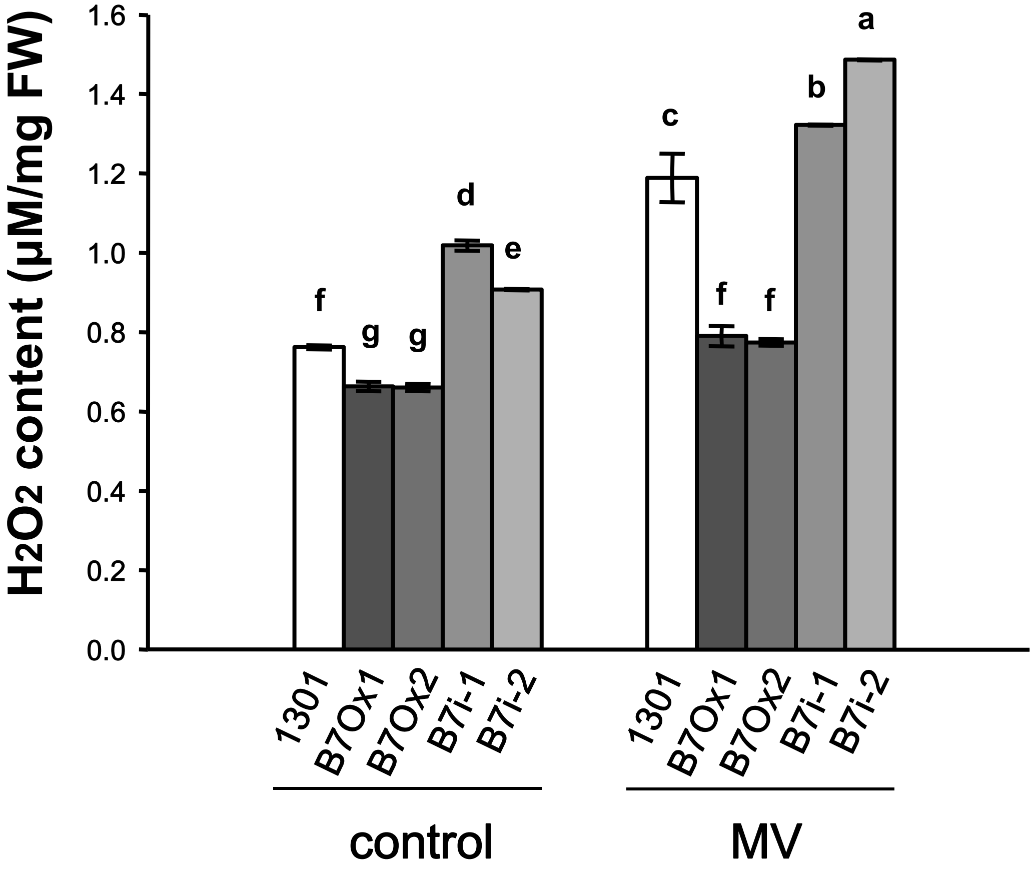

Supplementary Fig. S8. H_2O_2 content. Ten-day-old seedlings were treated with 10 µM MV for 24 h and intracellular H_2O_2 contents were measured using Amplex® Red Hydrogen Peroxide/Peroxidase assay kit. Letters indicate significantly ($P < 0.05$) different averages (Duncan's test).

Supplementary Table S2. Primers used for PCR and real-time PCR.

Primer ID	Sequence	Product (bps)
MSRB7-FE	GGAATTCATGGCAGCAATGACCG	450
MSRB7-RBg	GAAGATCTTTATTGGGAGGAACC	
B7-GW-RNAi-F2	AAAAAGCAGGCTATACCCCGACCCATTTTC	171
B7-GW-RNAi-R2	AGAAAGCTGGGTGCTGCCATGACGGGAATAG	
MSRB7-qF1	GATCTGTGTGAGCTTGCTAGG	120
MSRB7-qR1	GAGAGTACATCGAACGAAGTGG	
GSTF2/3-GW-F1	AAAAAGCAGGCT TCATGGCAGGTATCAAAG	693
GSTF2-GW-R2	AGAAAGCTGGGT CTCACTGAACCTTCTCGGAAG	
GSTF3-GW-R2	AGAAAGCTGGGT CTCACAGAACCTTCTCTG	693
GSTF8-GW-F1	AGAAAGCTGGGT CCTACTGCTTCTGGAGGTC	795
GSTF8-GW-R2	AAAAAGCAGGCT TCATGGGAGCAATTCAAGC	
GSTF2-qF1	CCAGCTTCCGAGAAGGTTCAGTG	192
GSTF2-qR1	GCCAAAGATACTCTCAAGAGCATACAC	
GSTF3-qF1	CAAGTAAATAAGCGTCTGCCTCATTG	97
GSTF3-qR1	ACGTACACACACTCACATAAGCTGAAACA	
GSTF8-qF1	GTGGGTAGTGGGTGGTGGTGAG	186
GSTF8-qR1	GGCCATGATGATCGAAGAAGAAGAA	
Actin2-qF1	ATTCAGATGCCCAGAAGTCTTGTTC	201
Actin2-qR1	GCAAGTGCTGTGATTTCTTTGCTCA	
B7pro-GW-F1	AAAAAGCAGGCTCATGTTATCAAAGAATTATGATCTACG	2035
B7pro-GW-R1	AGAAAGCTGGGTGACGGGAATAGGATTTG	
EF1α-qF1	GAGCCCAAGTTTTTGAAGA	113
EF1α-qR1	CTAACAGCGAAACGTCCCA	
18S-qF1	AAACGGCTACCACATCCAAG	154
18S-qR1	CCTCCAATGGATCCTCGTTA	
GSTF2-M100L-F2	GCAATCCTCGCCATTGGAATGC	
GSTF2-M104L-F3	GCAATCATGGCCATTGGACTCC	
GSTF2-M100/104L-F4	GCAATCCTCGCCATTGGACTCC	
GSTF3-M100L-F2	CAGTATGCAATCCTCTCCATTGG	
